# Spatially organized cellular communities shape functional tissue architecture in the pancreas

**DOI:** 10.1126/sciadv.adx5791

**Published:** 2025-11-12

**Authors:** Alejo Torres-Cano, Jean-Francois Darrigrand, Gabriel Herrera-Oropeza, Georgina Goss, David Willnow, Anna Salowka, Siwanart Ma, Debashish Chitnis, Morgane Rouault, Alessandra Vigilante, Francesca M. Spagnoli

**Affiliations:** ^1^ Centre for Gene Therapy and Regenerative Medicine, King’s College London, Great Maze Pond, London SE1 9RT, UK.; ^2^ Centre for Neurodevelopmental Biology, Institute of Psychiatry, Psychology & Neuroscience, King’s College London, London SE1 1UL, UK.; ^3^ 10x Genomics, Pleasanton, CA, USA.; ^4^ Hub for Applied Bioinformatics (HAB), King’s College London, Newcomen St., London SE1 1UL, UK.

## Abstract

Organ function depends on the precise spatial organization of cells across multiple scales, from individual units to cellular communities that form local niches and, ultimately, higher-order structures. Although cell identities are increasingly well defined, the spatial arrangement and interactions among diverse cell types remain poorly understood. Here, we combine single-cell and spatial transcriptomics to map pancreatic cell populations across space and time, from embryonic development to adult homeostasis in mice. Using these maps, we resolve spatial heterogeneity among pancreatic cell types and uncover epithelial-mesenchymal units as basic tissue niches, which we functionally characterize in both mouse and human models. We also demonstrate that the mesenchymal lineage diversifies into various specialized subtypes during development, but this complexity diminishes over time, ultimately converging into a few fibroblast subtypes in adulthood. Together, our findings reveal how different progenitor lineages codevelop and organize into structured communities that establish a functional pancreas, providing a framework to guide in vitro organogenesis and tissue engineering for pancreatic diseases.

## Teaser

Spatial maps reveal dynamic epithelial-mesenchymal niches shaping pancreatic development and function.

## INTRODUCTION

Organs are composed of multiple cell types that develop in concert and organize into distinct tissue architectures, following a hierarchical spatial arrangement that is essential for their specialized functions (*1*, *2*). Recent systematic single-cell RNA sequencing (scRNA-seq) efforts have provided in-depth insights into the cellular composition of most embryonic and adult tissues (*3*, *4*). However, the lack of spatial context in these analyses limits our understanding of how individual cells interact and arrange themselves to form and maintain functional tissue architectures. To address these questions, we need to shift our focus from individual cells to cellular communities on a collective scale. Functional tissue units (also known as niches) arise from the dynamic interactions and coordinated actions of all constituent cells, requiring a holistic approach that encompasses the entire tissue topology (*5*). Furthermore, resolving the spatial organization of different progenitor cells and their microenvironment, as well as understanding how this organization changes during development, will provide insights into the mechanisms that regulate cell fate specification and differentiation. This will ultimately help linking cellular position to the emergence of distinct cell identities.

Here, we focus on the pancreas to interrogate the multicellular-scale mechanisms that define functional tissue architectures during organ development. The pancreas is an organ with both exocrine and endocrine functions, which are fulfilled by different cell types organized in distinct arrangements and locations across the tissue (*6*). Both exocrine and endocrine units arise from one common pool of endodermal progenitors, which progressively undergo differentiation and arrange into their respective structural and functional organization, either duct networks connected to acini or endocrine clusters (also known as islets) (*6*, *7*). These spatial arrangements are driven by multicellular interactions among pancreatic epithelial cells and the surrounding microenvironment, which is composed of a mix of different cell types, including mesenchymal, endothelial, immune cells, and a rich extracellular matrix (ECM) (*8*–*11*). The mesenchyme represents the most abundant component of the pancreatic microenvironment, displaying remarkable heterogeneity at the transcriptomic level and, to some extent, distinct functions and embryonic origin (*9*, *11*–*13*). To date, the hierarchy and rules of interactions between these different cellular components that eventually result in emergent properties of tissue structure and function as pancreas development proceeds are yet to be defined. We hypothesize that an accurate cartography of the cell types populating the embryonic pancreas would illuminate uncharacterized interactions, as well as determine the modules responsible for establishing and maintaining its functional architectures.

In this study, we integrated single-cell and spatial transcriptomic analyses to comprehensively characterize the molecular and spatiotemporal heterogeneity of the developing mouse pancreas, with a particular focus on the poorly defined mesenchyme. Our results reveal that the pancreatic mesenchyme is made up of transcriptionally distinct subpopulations, each occupying distinct spatial domains within the developing pancreas. We further delineated the functional properties of these niches using both mouse and human model systems. Together, our findings offer insights into how progenitor cells of different origins (pancreatic endoderm and mesoderm) codevelop and organize into structured cellular communities that control cell fate decisions and guide the formation of a mature, functional pancreas.

## RESULTS

### High-resolution map of the mouse embryonic pancreas

**Fig. 1.**
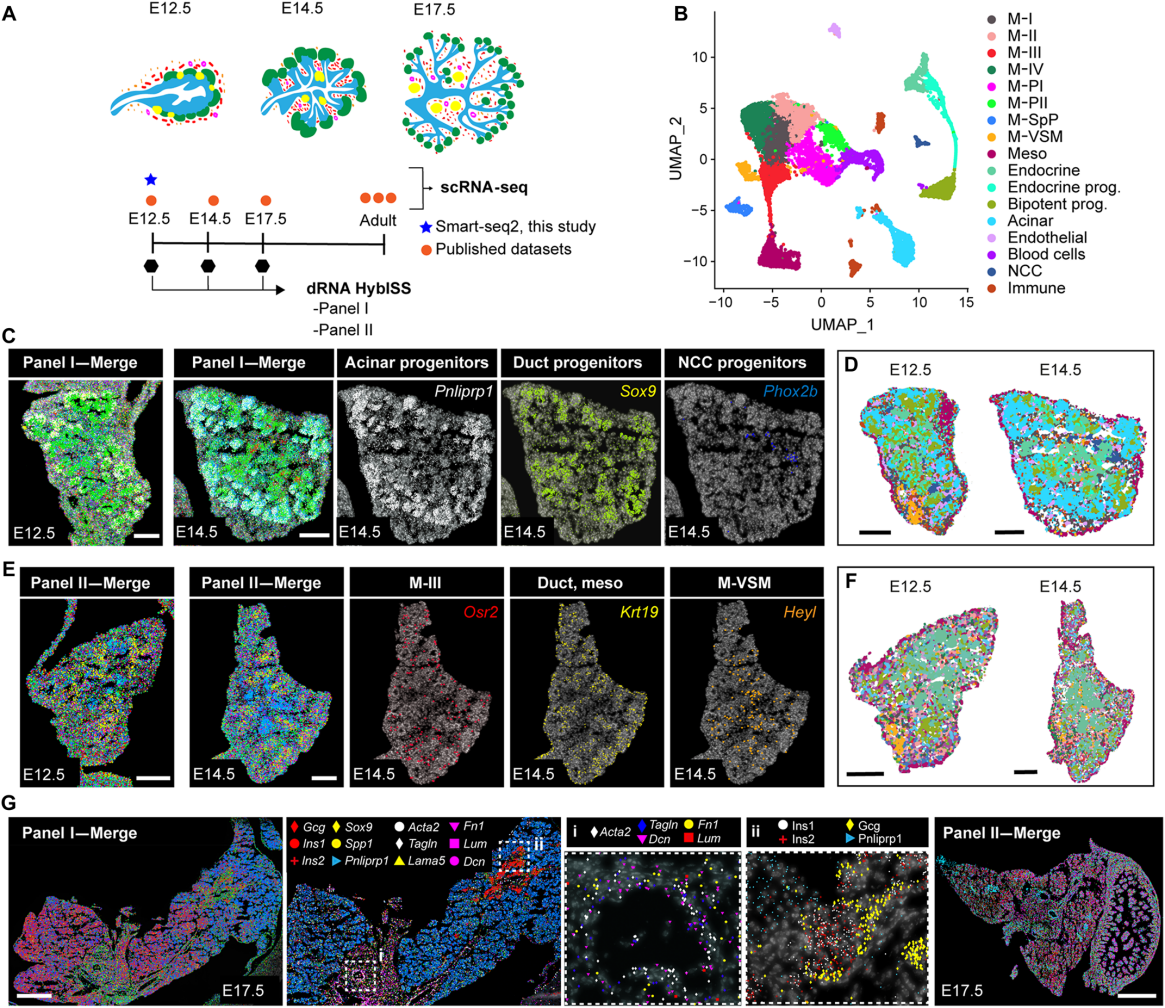
Major cell types and their spatial organization in the mouse embryonic pancreas as revealed by HybISS. (**A**) Overview of the experimental design and sample collection strategy. HybISS experiments were performed using two gene panels (panels I and II) targeting distinct sets of cell types in the embryonic pancreas. scRNA-seq datasets from embryonic pancreatic tissue at the indicated stages and adult tissue were used for gene panel selection and subsequent analysis. (**B**) Uniform Manifold Approximation and Projection (UMAP) plot showing clustering of scRNA-seq profiles of embryonic pancreatic tissue at E12.5, E14.5, and E17.5. Publicly available scRNA-seq datasets (*17*) were integrated with high-coverage Smart-seq2 that we performed on fluorescence-activated cell sorting (FACS)–isolated green fluorescent protein–positive (GFP^+^) cells from E12.5 pancreas of transgenic (Tg) *Prox1*-GFP, *Nkx2.5*-Cre;*R26*mTmG, and *Nkx3.2*-Cre;*R26*mTmG Tg embryos (see fig. S1). Clusters are color coded to indicate their annotated cell type. (**C**) Representative HybISS images showing all panel I marker genes (merge) or selected markers of distinct progenitor types in the pancreas at E12.5 and E14.5. Scale bars, 100 μm. (**D**) SSAM annotated cell maps of E12.5 and E14.5 panel I sections. Colors represent single-cell clusters as in (B). Scale bars, 100 μm. (**E**) Representative HybISS images showing all panel II marker genes (merge) or selected markers of distinct progenitor types in the pancreas at E12.5 and E14.5. Scale bars, 100 μm. (**F**) SSAM annotated cell maps of E12.5 and E14.5 panel II sections. Colors represent single-cell clusters as in (B). Scale bars, 100 μm. (**G**) Representative HybISS images of panel I and panel II marker genes in E17.5 pancreas. Close-ups of selected probe genes and their spatial distribution in the tissue are shown in (i) and (ii) dashed boxes. Scale bars, 500 μm.

To visualize the distribution of the different cell types that constitute the embryonic pancreas and define the cellular interactions underlying its spatial organization, we generated a high-resolution transcriptomic map using direct RNA hybridization-based in situ sequencing (dRNA HybISS; Cartana part of 10x Genomics) (*14*), which enables multiplexed transcript detection at single-cell resolution (Fig. 1A). Specifically, we applied the HybISS technology to mouse pancreatic tissue at three embryonic days 12.5 (E12.5), E14.5, and E17.5 (Fig. 1A). Dorsal and ventral embryonic pancreata were collected from multiple independent embryos and cryosectioned at different positions along the anterior-posterior axis. We generated two custom panels of probes to map the expression of genes associated with microenvironmental cell types based on clustering annotation in our scRNA-seq datasets, together with known pancreatic cell type markers (Fig. 1, A and B, fig. S1, and table S1).

Both panels included markers of major epithelial cell types from the pancreas: acinar, bipotent endocrine-duct, and endocrine progenitors (Fig. 1A and fig. S1). In addition, panel I comprised probes for endothelial, immune, neural, vascular, mesothelial, and mesenchymal cell types (Fig. 1, A, C, and G), while panel II was used to explore mesenchymal cell heterogeneity (Fig. 1, A, E, and G). We focused on the mesenchyme, as it represents the most abundant component of the pancreatic microenvironment and is required throughout pancreas development for organ growth and cell differentiation (*9*, *11*, *15*, *16*). Rather than being a uniform cellular population, pancreatic mesenchymal cells display heterogeneity at the transcriptomic level and, to some extent, distinct embryonic origin (*17*, *18*). Consistently, we previously reported two subsets of mesenchymal cells (Nkx2.5- and Nkx3.2-descendant cells), showing left-right asymmetry along the embryonic pancreatic tissue and distinct functions (*19*). Thus, with the HybISS analysis, we further explored the link between transcriptomic heterogeneity, the spatial location of pancreatic mesenchymal cells, and associated function.

To fully assess heterogeneity within the pancreatic mesenchyme, we integrated published scRNA-seq datasets (*17*) of mouse embryonic pancreatic cells with a high-coverage Smart-seq2 scRNA-seq that we performed on fluorescence-activated cell sorting (FACS)–isolated green fluorescent protein–positive (GFP^+^) cells from *Nkx2.5*-Cre;*R26*mTmG and *Nkx3.2*-Cre;*R26*mTmG transgenic (Tg) pancreatic mesenchyme and Tg[Prospero homeobox 1 (Prox1)-GFP] pancreata (Fig. 1A and fig. S1, A to J). Clustering identified eight mesenchymal clusters (M) with distinct gene expression profiles in line with previous studies (*17*). Two mesenchymal clusters were characterized by the expression of proliferative genes (*Mki67* and *Top2a*) (referred to as M-PI and M-PII); one cluster showed a gene signature (*Acta2* and *Tagln*) typical of vascular mesenchymal cells (M-VSM); one cluster was enriched with splenopancreatic mesenchymal genes, such as *Nkx2.5* and *Tlx1* (M-SpP) (*19*, *20*); and one separate cluster expressed hallmark mesothelial genes (*Upk3b* and *Wt1*) (*21*). Three additional mesenchymal clusters (M-I, M-II, and M-III) displayed specific marker gene sets of a less-characterized identity (fig. S1, C and H, and table S2).

Pancreatic samples at different time points were processed for dRNA HybISS concomitantly in the same run to minimize batch effects (Fig. 1A). Of the 96 gene probes present in the two panels, we successfully detected the expression of 94 of them (table S3). HybISS-based mRNA localization obtained in each dataset was used to generate spatial cell maps following two independent and complementary approaches: (i) SSAM (spot-based spatial cell type analysis by multidimensional mRNA density estimation) segmentation-free method (*22*) and (ii) segmentation-dependent method based on nuclear 4′,6-diamidino-2-phenylindole (DAPI) staining (*23*–*25*) (Fig. 1, D and F, and fig. S1K). Overall, both analyses successfully constructed spatial cell maps, which contained all cell types identified by scRNA-seq, and enabled us to chart the detailed spatial relationships between these cell types and to recognize multicellular pancreatic tissue features (Fig. 1, C to G, and fig. S2). Notably, cell type maps generated were comparable across all tissue sections of E12.5 and E14.5 panel I and panel II datasets (fig. S2).

Next, we used Tangram to align HybISS spatial profiling with scRNA-seq profiles (Fig. 1B) and impute spatial gene expression of those genes that were not included in the HybISS panels. We first assessed that a set of reference pancreatic genes (e.g., *Rbpjl*, *Nkx6.1*, and *Chgb*) recapitulated the expected gene expression patterns. Then, we assessed the spatial distribution of genes expressed by different mesenchymal populations, such as M-II (*Gap43*, *Syt6*, and *Epha4*), M-III (*Col14a1*), and M-PII (*Ccnb2* and *Cenpa*) (fig. S3A). Overall, this approach allowed for a more comprehensive analysis of gene expression patterns within a spatial context, effectively expanding our cellular mapping.

In addition, we examined how the mesenchymal subpopulations we have identified relate to previously published studies (*17*, *26*) (fig. S3, B to E). We observed substantial overlap in molecular signatures across datasets, particularly regarding the mesothelium, proliferative mesenchyme, and M-VSM (fig. S3, D and E). Further cross-dataset comparisons are needed to refine the existing classifications and establish a standardized nomenclature for pancreatic mesenchymal populations.

### Mesenchymal cells display heterogeneous spatial organization across the embryonic pancreatic tissue

**Fig. 2.**
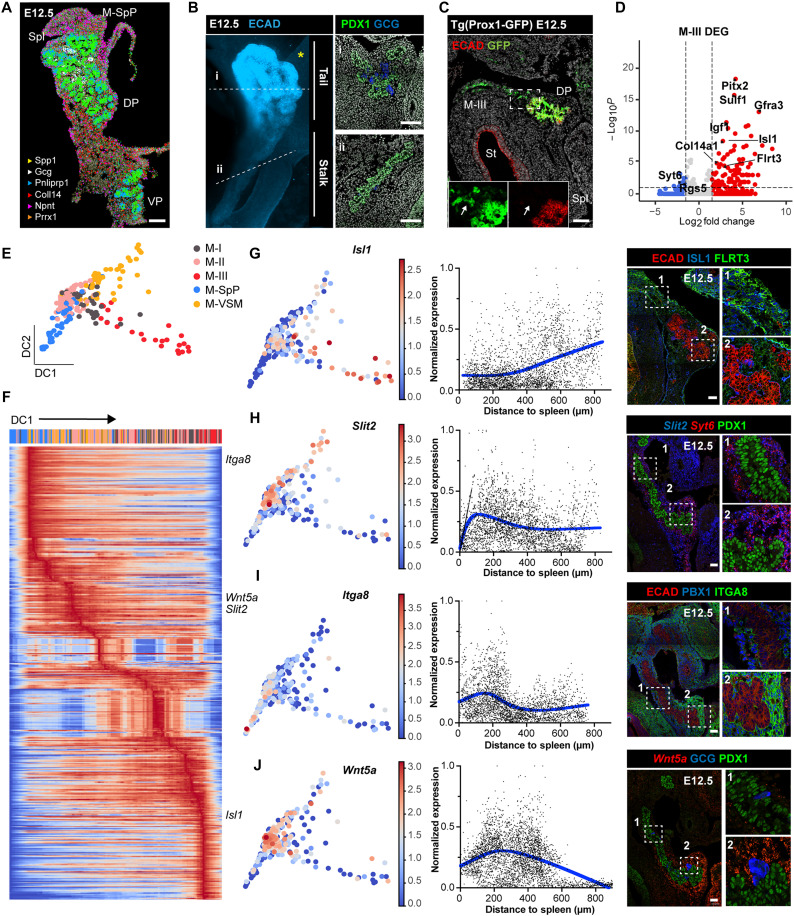
Heterogenous spatial organization of pancreatic progenitors and mesenchymal cell types across the developing pancreas. (**A**) Representative HybISS image showing selected genes from panel I in DP and VP at E12.5. Scale bar, 100 μm. (**B**) Representative three-dimensional (3D) rendering of light-sheet fluorescent microscopy image (left) and confocal microscopy images (right) of E12.5 pancreas stained with indicated antibodies. Right: Confocal IF images show transverse cryosections of DP at tail (i) and stalk (ii) levels. Hoechst was used as nuclear counterstain. Scale bars, 100 μm. Asterisk indicates approximate position of the spleen. (**C**) Representative confocal image of E12.5 Tg(*Prox1*-GFP) DP cryosection stained with antibodies against GFP and E-cadherin (ECAD). Arrows in the insets indicate Prox1-GFP^+^ M-III mesenchymal cells. St, stomach; Spl, Spleen. Scale bar, 100 μm. (**D**) Volcano plot showing differentially expressed genes (DEGs) between M-III and the other mesenchymal subpopulations. Selected top-ranked DEGs are highlighted. (**E**) Diffusion plot showing mesenchymal scRNA-seq profiles from E12.5 Smart-seq2dataset, colored by clusters as in fig. S1A. (**F**) Heatmap showing normalized and log-transformed expression levels of marker genes in the mesenchyme clusters along DC1. Genes of interest are indicated to the right. (**G** to **J**) Diffusion plots showing normalized and log-transformed expression levels of indicated subtype markers (left) in mesenchymal clusters and their validation by IF and RNAscope assays on E12.5 DP cryosections (middle and right). Fibronectin Leucine Rich Transmembrane Protein 3 (FLRT3) (G) was used as an additional marker for M-III subtype, *Syt6* (H) for M-II subtype, and PBX Homeobox 1 (PBX1) (I) for broad DP mesenchyme (table S1). Fluorescence intensity (FI) was plotted against distance to the spleen. FI was measured with QuPath software, and values were corrected by linear normalization within each embryo. Blue lines represent smoothing spline curves fitting the data. Numbered dashed boxes indicate the IF magnified area shown on the right. GCG, glucagon. Scale bars, 50 μm.

First, we used the HybISS-based mRNA localization to map the spatial distribution of major cell types in the pancreas and explore higher-order tissue organization in the pancreatic rudiment and to define its borders with surrounding organs, such as the stomach and spleen. The pancreas forms from two distinct buds arising from the dorsal and ventral regions of the foregut endoderm, which later fuse in development to give rise to the mature organ (*6*, *9*). The footprint of the two embryonic rudiments can be found in the adult tissue, wherein the dorsal pancreatic bud (DP) gives rise to part of the head, the body, and the tail of the pancreas, whereas the ventral pancreatic bud (VP) to the uncinate process and the remainder of the head (*6*, *9*). DP and VP progenitor cells display some differences in their transcriptional profiles both in mice and humans (*27*, *28*). Here, we used HybISS spatial transcriptomics to map spatial gene expression in the DP and VP and their respective microenvironments (Fig. 2A). Within the epithelium compartment, both HybISS and immunofluorescence (IF) staining analyses showed a higher number of endocrine progenitors in DP compared to VP at E12.5 and E14.5 (fig. S4, A, C, and E), suggesting that cellular differentiation in the developing pancreas is not uniform from a spatial point of view. In the surrounding microenvironment, we found some differences limited to the mesenchyme and the spatial distribution of transcriptionally distinct mesenchymal subtypes (fig. S4, A and B). Specifically, transcripts expressed by the M-SpP (*Nkx2.5*, *Kazald1*, and *Npnt*) were mostly restricted to the tissue surrounding the DP, while other mesenchymal genes in our HybISS panels (e.g., *Prxx1* and *Pdgfrb*) were expressed around both rudiments at E12.5 (fig. S4, A and B). In addition, by HybISS and RNAscope in situ hybridization validation assay, we found differences in the expression of *Wnt5a*, a marker of the M-II subpopulation (fig. S1C and table S1), being highly enriched in the DP mesenchyme and almost undetectable in the VP mesenchyme at E12.5 and E14.5 (fig. S4, C and D).

Next, we focused on the DP, which can be subdivided into two main structural domains based on morphological landmarks: a thin epithelium domain connected to the duodenum (also known as stalk) and a branched multilayered structure (also known as tail) (Fig. 2B), which give rise to the gastric lobe and splenic lobe, respectively (*20*, *29*). To assess any difference in the spatial organization of distinct cell types between the two DP domains and in their local environments, we combined HybISS spatial transcriptomics with scRNA-seq and IF analyses. Within the epithelium compartment, we found a higher number of endocrine cells in the DP tail domain when compared to the stalk, as shown by glucagon mRNA and protein distribution (Fig. 2, B and J, and fig. S4E). This was mirrored by the spatial arrangement of the M-SpP and M-II [Collagen 14a1 (*Col14a1*)–negative and *Kazald1*-positive] mesenchymal subpopulations, lying next to the tail domain and between the pancreas and the spleen (fig. S4, A and B).

At the opposite side of the DP, in the stalk region closer to the duodenum, mesenchymal cells mostly corresponded to the M-III cluster, positive for Prox1, and enriched for *Islet 1 (Isl1*) and *Pitx2* transcription factors, growth factors [e.g., *Insulin-like growth factor 1 (Igf1*)], and ECM proteins (e.g., *Col14a1*) (Fig. 2, C and D, fig. S4A, and table S1).

To further characterize the major axes of transcriptional variation in our data, we decided to use diffusion maps, a nonlinear dimensionality reduction algorithm that orders cells along components associated with coherent gene expression patterns, preserving the underlying data structure (*30*). This method has been successfully applied to a variety of different contexts, including spatial problems (*30*). To avoid confounding factors coming from dataset integration, we focused on our deep-coverage Smart-seq2 dataset of the E12.5 mesenchyme (Fig. 2E and fig. S1B). We also removed proliferative (M-PI and M-PII) clusters to prevent cell cycle ordering from being included in the dimensionality reduction. A top component of variation placed the M-SpP and M-III subpopulations at opposite sides of the inferred trajectory (pseudospace axis), with M-I, M-II, and M-VSM lying in between (Fig. 2E), which closely matched the HybISS spatial data. Transcriptional variation along this diffusion component (DC1) inferred distinct patterns of gene expression across this axis, from M-SpP to M-III (referred to as splenic-gut axis) (Fig. 2F). We then used RNAscope and IF staining to spatially validate selected differentially expressed genes (DEGs) enriched in distinct mesenchymal subtypes on cryosections of pancreatic tissue. Specifically, *Isl1*, whose transcript was up-regulated along the first DC, displayed similar spatial distribution, being abundant in M-III cells, next to the stalk region of the pancreatic epithelium, and at low abundance closer to the spleen (Fig. 2G). On the other hand, *Slit2*, *Integrin alpha-8 (Itga8*), and *Wnt5a* were mostly expressed in mesenchymal cells surrounding the DP tail closer to the spleen (Fig. 2, H to J). These results underscored differences in the spatial organization of the mesenchyme around the pancreatic epithelium (along both dorsal-ventral and splenic-gut axes), suggesting that microenvironmental signals from the mesenchyme are responsible for the nonuniform cellular differentiation observed in the developing pancreatic epithelium.

### Topographic maps reveal dynamic endocrine and exocrine pancreatic niches

**Fig. 3.**
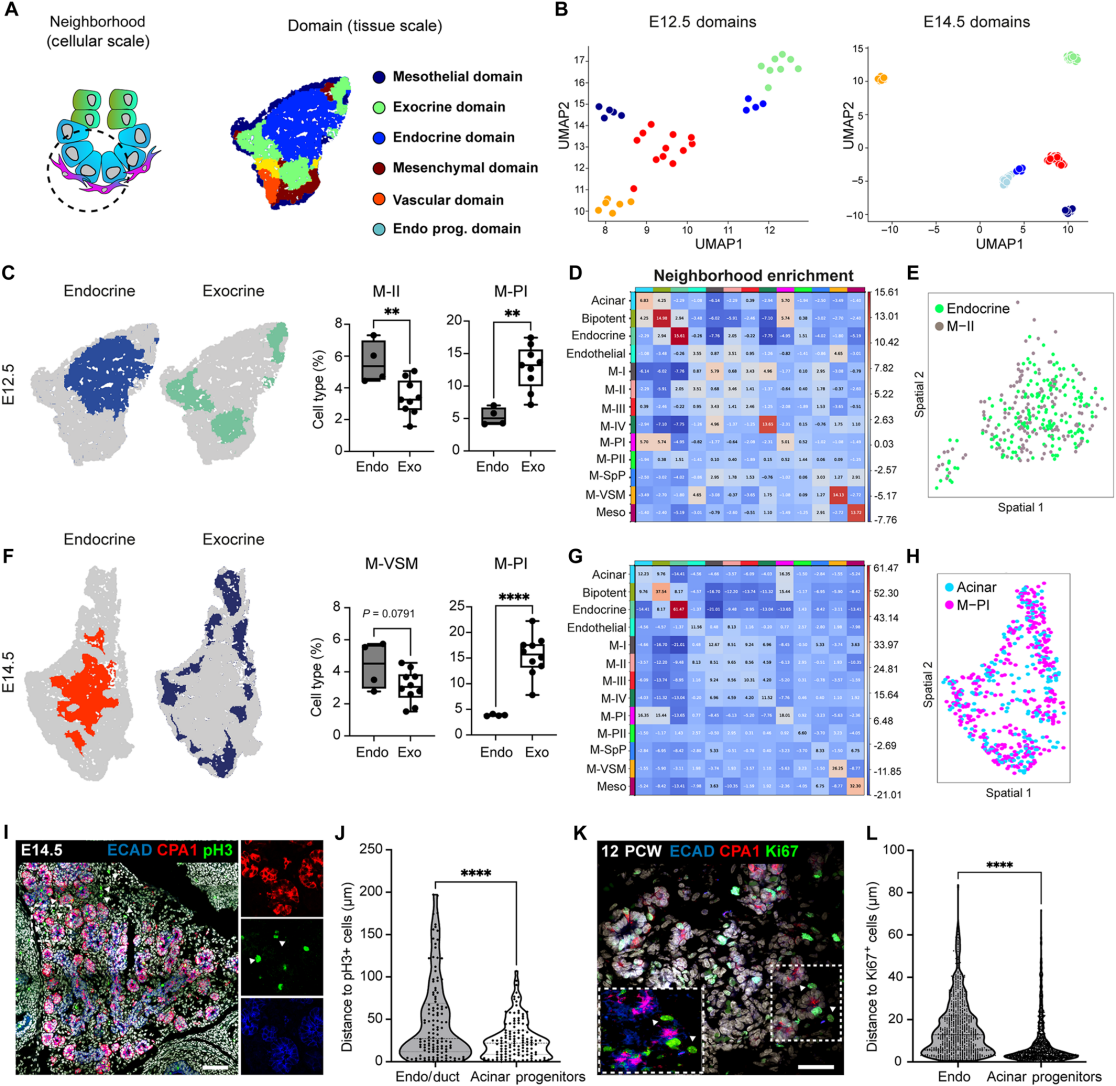
Tissue-domain analysis reveals mesenchyme niche-specific populations. (**A**) Schematics of the spatial analysis frameworks: At cellular scale (left), spatial neighborhoods encompassing the 10 closest cells around each cell were used to calculate cluster pair neighborhood enrichment; at tissue scale (right), tissue areas with similar local cell type composition were clustered to identify tissue domains. (**B**) UMAPs showing E12.5 and E14.5 specific tissue domains as identified by SSAM on panel II tissue image datasets. Cellular composition in each tissue domain is included in table S4. Color code is as in (A). (**C**) Representative endocrine and exocrine tissue domain maps from E12.5 pancreas (left) and quantification of indicated mesenchyme subpopulations in each domain (right). **P < 0.01, two-tailed unpaired *t* test. (**D**) Neighborhood enrichment score between cell types in E12.5 pancreas (*n* = 9 tissue sections). Positive enrichment indicates proximity between cell types. (**E**) Spatial distribution of indicated cell types on HybISS-based map of E12.5 pancreas. (**F**) Representative endocrine and exocrine tissue domain maps from E14.5 pancreas (left) and quantification of indicated mesenchyme subpopulations in each domain (right). ****P < 0.0001, two-tailed unpaired *t* test. (**G**) Neighborhood enrichment score between cell types in E14.5 pancreas (*n* = 10 tissue sections). (**H**) Spatial distribution of indicated cell types on HybISS-based map of E14.5 pancreas. (**I**) Representative confocal image of E14.5 pancreas stained with antibodies against the indicated markers. Hoechst was used as nuclear counterstain. Scale bar, 100 μm. (**J**) Distance between phosphorylated histone H3–positive (pH3^+^) proliferating mesenchymal cells and E-cadherin^+^/CPA1^+^ acinar progenitors or E-cadherin^+^/CPA1^−^ endocrine/ductal cells. *n* = 125 cells; three embryos. ****P < 0.0001, two-tailed unpaired *t* test. (**K**) Representative confocal image of 12 PCW human pancreas stained with antibodies against the indicated markers. Scale bar, 50 μm. (**L**) Distance between Ki67^+^ proliferating mesenchymal cells and E-cadherin^+^/CPA1^+^ acinar progenitors or E-cadherin^+^/CPA1^−^ endocrine/ductal cells. *n* = 748 (endocrine); *n* = 1266 (acinar) cells. ****P < 0.0001, two-tailed unpaired *t* test.

Next, we sought to use HybISS spatial transcriptomics to resolve spatial organization within the pancreatic tissue at the cellular level, including the relative positioning of different cell types and relationships among them. To this aim, we carried out two separate but complementary approaches at mesoscale (tissue domain) or microscale (cell neighborhoods) levels of organization, leveraging segmentation-free and segmentation-based frameworks, respectively (Fig. 3A).

The SSAM framework enabled us to define distinct pancreatic tissue domains by clustering local cell type composition from each sample in both E12.5 and E14.5 panel I and panel II (Fig. 3, A and B, fig. S2, and table S4). In panel I, we identified five different tissue domains at E12.5 and E14.5 (fig. S5, A and B), which were named on the basis of the predominant cell types in each domain. Similarly, the domains in the panel II dataset were classified as exocrine (i.e., acinar and bipotent endocrine-duct progenitors), endocrine, endocrine progenitor, mesothelial, mesenchymal, and vascular (Fig. 3, A and B, and table S4). At E14.5, we observed two separate endocrine clusters, one primarily enriched in endocrine progenitor cells and the other one in differentiated endocrine cells, indicating an increase in the complexity of the pancreatic tissue (Fig. 3B). The cellular composition of the domains revealed a differential local enrichment of mesenchymal subtypes. For instance, at E12.5, M-PI was enriched in the exocrine domain, whereas M-II was more abundant in the endocrine domain (Fig. 3C). At E14.5, we observed the same enrichment of M-PI cells in the exocrine domain and a tendency for M-VSM to be enriched in the endocrine domain (Fig. 3F). To refine the granularity of the tissue domain analysis at the microscale and validate its findings, we performed a neighborhood enrichment analysis on our segmented tissue maps (fig. S2E). Consistently, M-PI was found enriched in proximity to the acinar progenitors, whereas M-II was the only mesenchymal subtype with a positive enrichment value in the endocrine neighborhood (Fig. 3, D to H). Neighborhood enrichment analysis also validated the proximity between neural crest cells (NCC) and endocrine cells from panel I (fig. S5, C to E). Last, we validated the predicted spatial proximity between proliferative mesenchyme subtype and acinar cells by IF staining and quantitative measurement of the distance between mesenchymal cells positive for the mitotic marker phosphorylated histone H3 (pH3) and acinar progenitor marker Carboxypeptidase A1 (CPA1) (Fig. 3, I and J, and fig. S5H). M-II enrichment in the endocrine domain was also corroborated by IF staining for the M-II marker GAP43, which showed preferential distribution in the central “core” plexus of the pancreas, where endocrinogenesis occurs (*11*). By contrast, M-I and M-III [Igf1 and SPARC Related Modular Calcium Binding 2 (SMOC2)] markers were enriched at the periphery of the pancreatic epithelium (fig. S5, F and G).

Next, to start assessing the extent of conservation of the distinct mesenchyme subpopulations and their spatial organization in humans, we first combined our integrated mouse scRNA-seq dataset with a recently published human dataset (*27*) from human fetal pancreas at equivalent development stages (fig. S6, A and B). The resulting dataset showed a clear resemblance between a subset of human mesenchyme clusters and mouse subpopulations, as well as conservation of relevant markers, ligands, and ECM components in human cells (fig. S6, C and D).

The combined dataset was used throughout the study to assess similitudes and differences between mouse and human populations. In addition, to characterize the conservation of the acinar–M-PI spatial proximity, we performed IF analyses on human fetal pancreatic tissue at 12 postconception weeks (PCW) using a similar set of antibodies to visualize proliferating cells in the mesenchyme (E-cadherin^−^) and CPA1^+^ cells (Fig. 3K). Quantitative measurement of cell-to-cell distances showed significant proximity between proliferating mesenchymal cells and acinar progenitors in human fetal pancreatic tissue, similar to that in the mouse (Fig. 3L). Overall, our spatial analysis unveiled differential multicellular composition around endocrine and exocrine progenitor cells, highlighting mesenchymal niches with possibly distinct functional supporting roles around the two main pancreatic units.

### M-II signals and secreted ECM components regulate endocrine differentiation

**Fig. 4.**
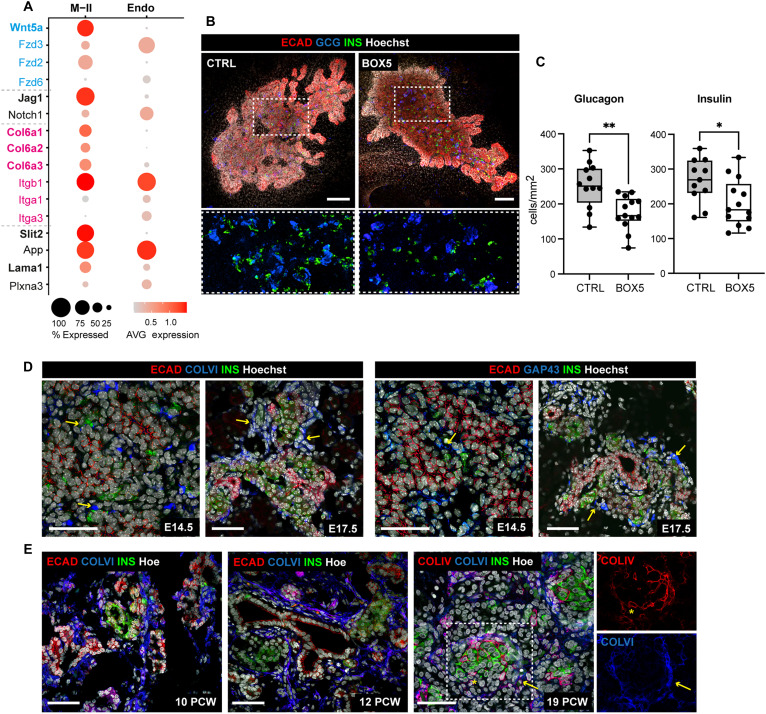
M-II signaling niche promotes endocrinogenesis. (**A**) Dot plot showing ligands and cognate receptors expression in M-II and endocrine cells. The analysis is based on the high-coverage, Smart-seq2 dataset. Ligand and receptor pairs are color coded, ligands shown in bold font and receptors in regular font. Color bar indicates the linearly scaled mean of expression level. AVG, average. (**B**) Representative whole-mount IF images of mouse pancreatic explants treated for 2 days with BOX5 or left untreated as controls (CTRL), stained with antibodies against E-cadherin (ECAD), glucagon (GCG), and insulin (INS). Hoechst dye was used as nuclear counterstain. Bottom: Higher magnification of the boxed regions. Scale bars, 150 μm. (**C**) Quantification of glucagon^+^ and insulin^+^ cells in BOX5-treated and untreated pancreatic explants. Cell counts were normalized to the average E-cadherin^+^ epithelium area (in square millimeters). *n* = 11 to 13 explants per condition. *P < 0.05 and **P < 0.01, two-tailed paired *t* test. (**D**) Representative confocal images of E14.5 and E17.5 pancreatic cryosections immunostained for insulin and the M-II markers Collagen VI (COLVI) or GAP43. Arrows indicate proximity of M-II markers and insulin^+^ cells. Hoechst dye was used as nuclear counterstain. Scale bars, 50 μm. (**E**) Representative confocal images of human fetal pancreatic tissue, between 10 and 19 PCW, stained with antibodies against indicated markers. Right: Single-channel magnifications of the boxed region in 19 PCW. Arrow and asterisk indicate COLIV and COLVI deposition, respectively, around insulin^+^ clusters. Scale bars, 50 μm.

To identify cell-cell signaling events that might regulate pancreatic endocrine and exocrine development, we assessed the main signaling signatures across the pancreatic mesenchyme and used CellChat (*31*) to probe ligand-receptor (L:R) signaling events enriched between the identified mesenchyme subtypes and different pancreatic progenitor populations (Fig. 4A and fig. S7, A and B). This highlighted a set of signaling pathways that might be implicated in pancreatic mesenchyme diversification; a subset of these patterns was experimentally validated (fig. S7, B to F). For instance, receptor tyrosine kinase/extracellular signal–regulated kinase (ERK) signaling was found to be mainly released by the mesothelium (source) and received by M-SpP, M-II, and M-PI (targets) (fig. S7, C and D). The transcriptome analysis of pancreatic tissue from fibroblast growth factor 9 (Fgf9) knockout (KO) mice (*32*) showed gene expression modulation in mesenchymal cells along the splenic-gut axis, from M-SpP to M-III (fig. S7, G and H). WNT signaling was primarily active in the epithelium (trunk progenitors), as evidenced by the quantification of lymphoid enhancer factor 1 (LEF1) downstream effector (fig. S7, D to F). On the other hand, heterogeneous nuclear active Yes-associated protein (YAP) expression was detected throughout the mesenchyme (fig. S7, C and D).

Next, given the spatial proximity identified between the endocrine and M-II cells, we focused on the cross-talk between these two populations (Fig. 4A and fig. S8A). Our predicted L:R interactions indicate strong intercellular communication between endocrine and M-II cells through the NOTCH signaling (*Notch1* and *Jag1*) and WNT signaling [*Wnt5a* and *Frizzled 3* (*Fzd3*)], as well as ECM molecules (*Lama1*, *Col6a1*, *Col6a2*, and *Col6a3*) and integrin receptors (Fig. 4A). Some of these cell-cell communication pathways have been studied for their role in endocrinogenesis, such as the NOTCH pathway (*33*, *34*), giving high confidence to our L:R predictions. In addition, components of the noncanonical WNT pathway have been previously involved in regulating endocrine cell differentiation (*35*–*37*), even if their spatiotemporal activities and cellular source have not been resolved. Our spatial analysis identified a localized expression of *Wnt5a* in the mesenchyme, mostly in the M-II subtype, next to the DP tail, which corresponded to the preferential site for endocrine cell differentiation (Fig. 2J and fig. S4, C and D). To functionally interrogate the role of WNT5A in the pancreatic mesenchyme, we used pancreatic explants dissected from E12.5 wild-type mouse embryos, as an epithelium-mesenchyme coculture system (*38*). Explants were cultured ex vivo for 48 hours in the presence of the WNT5A antagonist, BOX5, previously shown to attenuate WNT5A-mediated Ca^2+^ and c-Jun N-terminal kinase (JNK) signaling (fig. S8B) (*39*). We found that BOX5 treatment reduced the number of insulin- and glucagon-positive cells compared to nontreated control (CTRL) samples (Fig. 4, B and C). Consistently, the number of Neurogenin 3–positive endocrine progenitors was also decreased in BOX5-treated pancreatic explants (fig. S8, C and D). Together, these results are in support of a role for WNT5A in endocrine cell differentiation.

**Fig. 5.**
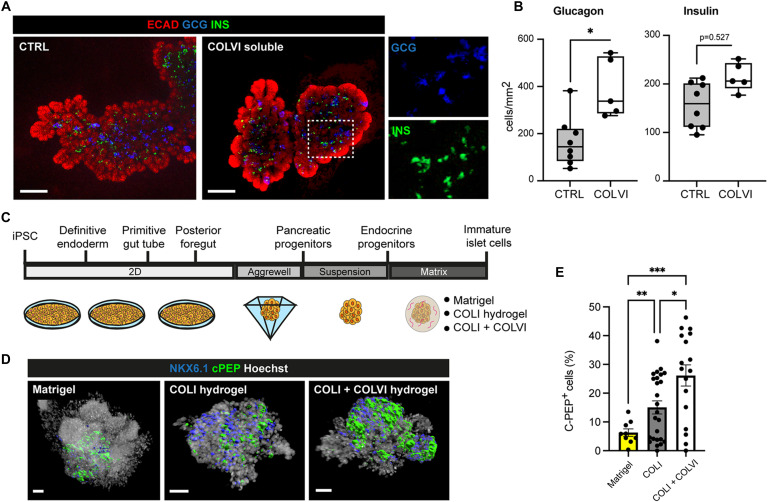
COLVI-enriched matrix exerts a conserved proendocrine function. (**A**) Representative whole-mount IF images of mouse pancreatic explants treated for 2 days with soluble COLVI or left untreated (CTRL), stained for E-cadherin, glucagon, and insulin. Right: Single-channels high magnification of the boxed regions. Scale bars, 150 μm. (**B**) Quantification of glucagon^+^ and insulin^+^ cells in COLVI-treated and untreated pancreatic explants. Cell counts were normalized to the average E-cadherin^+^ epithelium area (in square millimeters). *n* = 5 to 6 explants per condition. *P < 0.05, two-tailed paired *t* test. (**C**) Schematic of the iPSCs directed differentiation protocol into pancreatic β-like cells. iPSC-derived endocrine progenitor clusters were embedded in different matrices at day 18 and further cultured for 4 days. (**D**) Representative whole-mount IF images of differentiated endocrine clusters stained for NKX6.1 and C-peptide (C-PEP). C-PEP and NKX6.1 stainings were rendered as 3D surfaces using Imaris Surface tool. Hoechst was used as nuclear counterstain. Scale bars, 50 μm. (**E**) Quantification of C-PEP^+^ cells in differentiated iPSC-endocrine clusters; counts were normalized to the total cell number. *n* = 25 clusters for COLI, *n* = 17 for COLI + COLVI, and *n* = 9 for Matrigel. *P < 0.05, **P < 0.01, and ***P < 0.001, Brown-Forsythe and Welch analysis of variance (ANOVA) tests.

Next, we sought to investigate the ECM molecules that compose the M-II niche and their potential impact on pancreatic endocrine development. We primarily focused on Collagen VI (COLVI) because, despite being among the most abundant ECM molecules in the adult pancreatic islets (*40*), its function in the context of pancreatic development is unknown. We found that COLVI increases, both at the transcript (*Col6a1*, *Col6a2*, and *Col6a3*) and protein levels, during development (fig. S8, E and F), being densely distributed around the endocrine progenitors and insulin-positive cells in the mouse and human fetal pancreas, respectively (Fig. 4, D and E). To directly investigate the effects of COLVI on endocrine development, we treated mouse pancreatic explants with soluble COLVI for 2 days and then assessed cell differentiation. We observed a significant increase in the number of both insulin- and glucagon-positive cells upon COLVI treatment when compared to nontreated CTRL samples (Fig. 5, A and B). As additional approach, we embedded pancreatic explants in hydrogels containing different ECM components. COLVI was mixed with COLI, as it shows limitations in forming hydrogels on its own, to create interstitial-like ECM. Similar to the recombinant COLVI, the COLI/COLVI hydrogel led to an increase in the number of insulin-positive cells in the embryonic pancreatic culture (fig. S8, G and H). Similar effects were also observed in the COLI hydrogel but not in Matrigel, which mimics a basal membrane–like ECM, including mostly COLIV and laminins (fig. S8, G and H). This is in line with our systematic analysis of ECM-ECM and cell-ECM communication networks across the mesenchyme clusters and pancreatic epithelium (fig. S9). Applying Matricom (*41*) to our scRNA-seq datasets, we identified the endocrine progenitors as the cell type with the lowest expression of ECM-related genes, suggesting a greater reliance on an extrinsically provided, ECM-enriched niche (fig. S9). Furthermore, on the basis of the expression profiles of integrin-α subunits, our analysis suggests that endocrine cells preferentially interact with collagens via Integrin (Itga1-Itgb1) heterodimers, whereas the Itga5/Itga7-Itgb1 heterodimers act primarily as laminin receptors (*10*, *42*) (fig. S9F).

Next, to investigate the functional conservation of ECM effects on human endocrine differentiation, we used a modified human pluripotent stem cell culture system for modeling human pancreatic development (Fig. 5C). Specifically, induced pluripotent stem cells (iPSCs) were first differentiated to the pancreatic progenitor stage in two dimensions and then embedded at the endocrine progenitor stage in the same hydrogel composition used for the mouse explants and cultured for 5 days, until collection (Fig. 5D). In human cells, COLVI had a specific beneficial effect, promoting an increase in the number of insulin-positive cells as compared to the COLI alone and Matrigel hydrogels (Fig. 5, D and E). Together, our findings unveiled an endocrine niche, enriched for M-II mesenchyme, with conserved functional components in humans, such as COLVI.

### Embryonic Nkx2.5^+^ M-SpP progenitors give rise to distinct populations of adult pancreatic fibroblasts

**Fig. 6.**
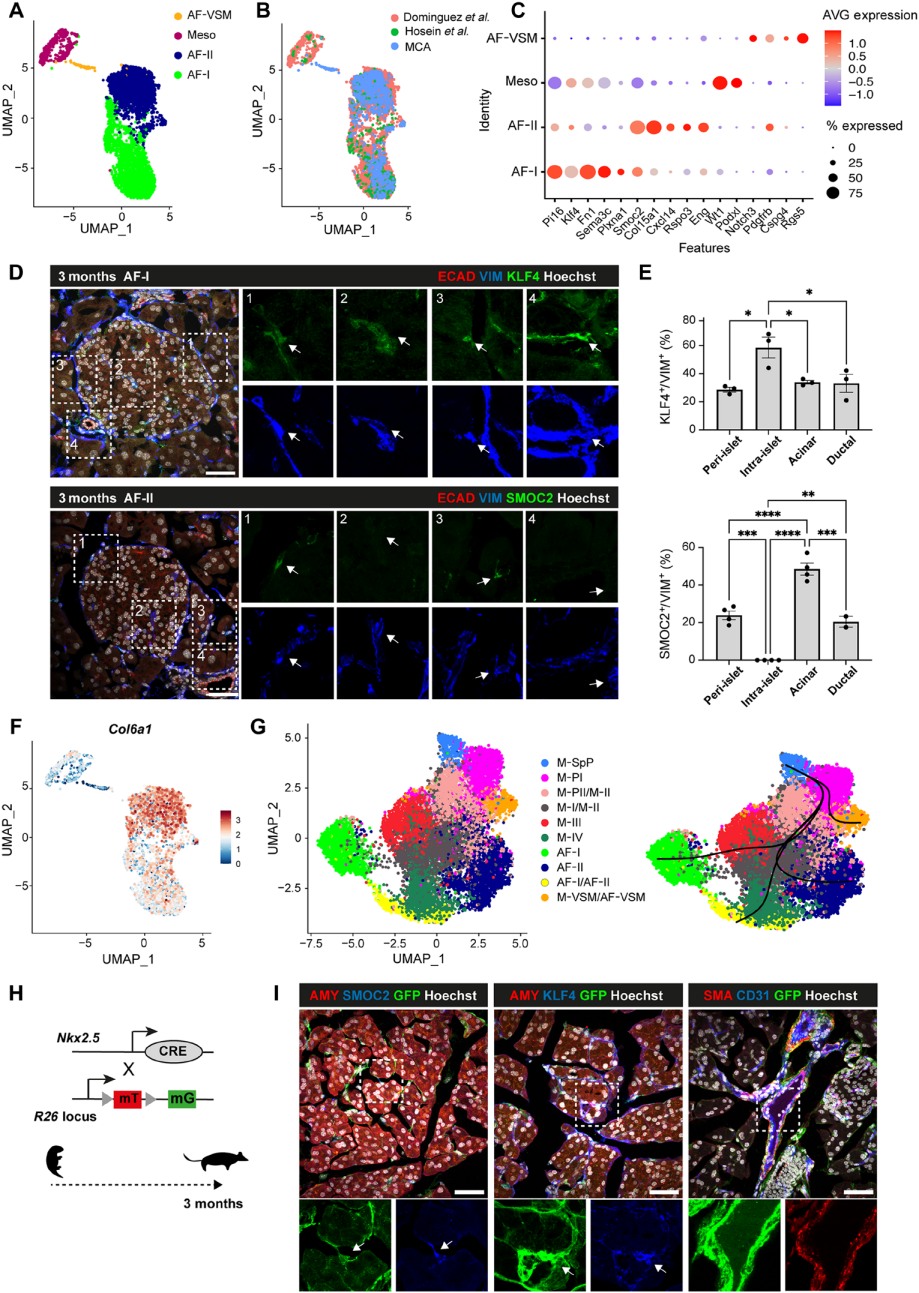
Embryonic Nkx2.5^+^ M-SpP lineage contributes to adult pancreatic fibroblasts. (**A** and **B**) UMAP visualization of adult pancreatic fibroblasts from three integrated scRNA-seq datasets (*3*, *47*, *48*). Clusters are color coded on the basis of cell type identity (A) or dataset (B). MCA, Mouse Cell Atlas. (**C**) Dot plot showing the expression of selected marker genes for each cell cluster from (A). Color bar indicates the linearly scaled mean of expression level (see table S5). (**D**) Representative images of adult pancreas cryosections stained with antibodies against the pan-mesenchymal marker vimentin (VIM) and AF-I–specific KLF4 and AF-II–specific SMOC2 markers. Right: Single-channels high magnification of the boxed regions. Arrows indicate AF-I (KLF4^+^) and AF-II (SMOC2^+^) cells in contact with epithelial structures (1, peri-islet; 2, intra-islet; 3, acinar; 4, ductal). Scale bars, 50 μm. (**E**) Quantification of the percentage (%) of SMOC2^+^ and KLF4^+^ cells per niche over total VIM^+^ cells. *n* = 3 to 4 pancreas. Error bars represent ± SEM. *P < 0.05, **P < 0.01, ***P < 0.001, and ****P < 0.0001, one-way ANOVA test. (**F**) Feature plot showing *Col6a1* expression in AF cells. Color bar represents mean log-transformed expression. Clusters are labeled on the basis of the annotation in (A). (**G**) Left: UMAP showing clustering of scRNA-seq profiles of mesenchymal cells from embryonic and adult pancreas integrated dataset. Clusters are labeled on the basis of the annotation of adult (A) and embryonic mesenchyme (Fig. 1B) datasets. Right: Slingshot-inferred lineages on embryonic–adult mesenchyme clusters, showing putative hierarchy of the pancreatic mesenchymal lineage. (**H**) Schematics of the lineage tracing strategy using *Nkx2.5*-Cre;*R26*mTmG mouse. (**I**) Representative images of IF staining on *Nkx2.5*-Cre;*R26*mTmG adult pancreas for the indicated markers. Bottom: Single-channel magnified views of the boxed areas. Arrows indicate *Nkx2.5*-Cre–descendant cells contributing to distinct AF populations. AMY, Amylase. Scale bars, 50 μm.

Mesenchymal progenitors give rise to mature adult tissue-resident fibroblasts, which support organ homeostasis and participate in fibrosis and diseases, such as cancer (*43*). Despite adult fibroblasts (AFs) playing a major role in pancreatic fibrosis and cancer progression and reactivating developmental programs at the injury site to some extent (*44*–*46*), their ontogeny and lineage relationships in the embryonic pancreas are still elusive. To answer these questions, we extended our analysis to adult tissue homeostasis. First, we integrated scRNA-seq datasets of healthy mouse pancreatic tissue from three different studies (*3*, *47*, *48*) (Fig. 6, A and B). After batch correction and clustering, we identified a mesothelial cluster [Keratin 18 (*Krt18*)], a vascular cluster (AF-VSM) [Actin Alpha 2, Smooth Muscle (*Acta2*^+^)] and two main AF clusters (Fig. 6, A and B). AF-I cells expressed genes that have been previously linked to reticular fibroblasts (i.e., *Klf4*, *Mfap5*, and *Cd34*) (*49*) and neuronal guidance-associated genes (*Sema3a* and *Plxna1*), while AF-II cells were characterized by a high level of expression of collagen and other ECM genes, including *Col6* genes, stellate cell marker, *Rspo3*, and high levels of *Smoc2* (Fig. 6, C to F).

Next, we quantified the distribution of both AF-I and AF-II in different niches of the adult pancreas. Both cell types were similarly represented in the acinar and ductal niches but showed differences with respect to the islets, being both at the islet periphery and only AF-I cells infiltrating them (Fig. 6, D and E).

Last, to understand the developmental origin and build a cellular taxonomy of the mesenchymal lineage from embryonic pancreas to adult homeostasis, we combined in silico analysis with in vivo genetic lineage tracing experiments. To better understand the hierarchy of the mesenchymal lineage, we first used partition-based graph abstraction (PAGA) (*50*) to generate a graphic representation of the relationships between the embryonic mesenchymal clusters (fig. S10). The PAGA graph laid the mesothelial cluster (*Nkx2.5*^−^) at one extreme of the graph, immediately connected to the M-III, then M-I, and M-VSM, following a trajectory previously delineated both in silico (*17*) and in vivo (*51*, *52*). At the opposite side of the graph, it was positioned the M-SpP cluster (*Nkx2.5*-high) directly connected to M-PI, M-PII, M-II, and M-VSM (fig. S10, A and B). Next, to trace Nkx2.5^+^ progenitors into adulthood and assess the possible trajectories and contributions of this lineage in the adult pancreatic mesenchyme, we first integrated embryonic and adult mesenchymal single-cell datasets (Fig. 6G). Then, we used Slingshot to obtain lineage trajectories and projected them into the Uniform Manifold Approximation and Projection (UMAP) representation (Fig. 6G). Using the M-SpP (*Nkx2.5^+^*) mesenchyme as root for the analysis, it uncovered different trajectories for the vascular cells (AF-VSM) and the two AFs. The lineage bifurcation of AFs started during embryonic development with M-III giving rise preferentially to AF-I cells and the other embryonic clusters to AF-II (Fig. 6G). Next, we used the *Nkx2.5*-Cre mouse Tg model with the *R26*-mTmG reporter line to track embryonic mesenchymal cells into adult tissue. To confirm that *Nkx2.5*-Cre specifically labels early pancreatic mesenchyme and is not active in the adult, we examined *Nkx2.5* expression in both scRNA-seq datasets and tissue samples. *Nkx2.5* transcript was barely detectable in pancreatic tissue sections at E14.5 and absent from E17.5 onward (fig. S10, A, C, and D). In line with the pseudotime model, our analysis revealed the presence of KLF Transcription Factor 4 (KLF4)^+^, GFP^+^ (AF-I), SMOC2^+^ GFP^+^ (AF-II), and Smooth muscle actin (SMA)^+^ GFP^+^ (AF-VSM) cells in the adult pancreas (Fig. 6, H and I), confirming the contribution of *Nkx2.5^+^* embryonic mesenchymal lineage to adult pancreatic fibroblasts.

## DISCUSSION

Our study provides a high-resolution spatiotemporal profiling of the developing pancreas, resolving the spatial organization of pancreatic cells and their surrounding microenvironment at a multiscale level. We identified potential mediating pathways for cell proximity effects at the organ scale, as well as the multicellular mechanisms that define spatial niches within the two main functional compartments of the pancreas: the endocrine and exocrine domains. Notably, these niches displayed differences in the mesenchymal subtype composition; the M-II was enriched next to endocrine progenitors, while the proliferative M-PI next to acinar progenitors. Our findings extend previous studies highlighting transcriptomic heterogeneity within the pancreatic mesenchyme (*17*, *18*, *26*, *27*, *53*, *54*), demonstrating that mesenchymal cells consist of several distinct, functional subtypes, distinguishable by their gene expression profiles, origin, and spatial location. Open questions remain about the mechanisms driving the transcriptional and functional diversity among mesenchymal/fibroblast subtypes. Although we identified signaling pathways establishing spatial gradients in the mesenchyme, further functional studies are needed to provide a better understanding of their role in mesenchymal cell fate diversification. Besides the impact of local signaling, fibroblast identity and behavior are influenced by their embryonic lineage (*55*). However, the developmental origin of the pancreatic mesenchyme is not fully understood. While a fate mapping study by 1,1′-dioctadecyl-3,3,3′,3′-tetramethylindocarbocyanine perchlorate labeling (*51*) and lineage tracing experiments (*52*) in the mouse embryo have shown that the mesothelium contributes to the pancreatic mesenchyme, our previous work (*19*), together with others (*11*, *13*), suggests that the mesenchyme arises from multiple sources. These include a subpopulation of the splanchnic mesoderm, marked by Nkx2.5, which gives rise to the M-SpP and is distinct from the mesothelium (*19*). Here, we further characterized Nkx2.5-lineage descendants and their contribution to adult pancreatic fibroblasts. We showed that *Nkx2.5* expression is down-regulated from E14.5 onward and becomes undetectable in the adult. These results support the use of *Nkx2.5*-Cre as a reliable driver for lineage tracing of M-SpP–derived cells throughout pancreatic development into adulthood. Our findings, together with other recent studies (*52*, *56*–*58*), indicate that the adult pancreatic stroma, as described for many other viscera, has a mixed origin from the splanchnic mesoderm [Nkx3.2^+^, Nkx2.5^+^, Isl1^+^, and Homeobox B6 (Hoxb6^+^) subpopulations] and Wilms’ tumor 1 Transcription Factor (WT1)-expressing mesothelium-derived cells. Notably, it has been shown that not all resident fibroblast populations expand upon injury and transition into cancer-associated fibroblasts within the pancreas (*56*–*58*). The potential role of Nkx2.5-descendant fibroblasts in the adult pancreatic homeostasis or disease states remains to be elucidated.

Beyond the pancreas, remarkable diversity and functional variety of mesenchymal cells and fibroblasts have been recognized across multiple organs, both between organs (interorgan) and within individual tissues (intraorgan) (*55*, *59*, *60*). Interorgan heterogeneity has become obvious from transcriptomics analyses and comparison of gene expression of murine and human mesenchyme and AFs from different tissues (tail, skin, lung, liver, heart, kidney, pancreas, and others) (*55*, *59*, *60*). These studies showed that AFs display organ-specific transcriptome signatures that reflect their embryonic origins, such as “lasting blueprint,” and that these signatures and their functional identity are maintained within coculture systems (*59*). In addition to tissue-specific features, a recent scRNA-seq fibroblast atlas identified two universal fibroblast subtypes, namely, Peptidase Inhibitor 16 (Pi16)^+^ and Col15a1^+^ subtypes, across multiple murine adult tissues (*61*). In our study, clustering of the integrated adult pancreas dataset identified two main fibroblast populations: Pi16^+^ (AF-I) and Col15a1^+^ (AF-II) clusters, alongside the AF-VSM and mesothelium populations. Nevertheless, both fibroblast clusters also express a distinct set of pancreas-specific markers that differentiate them from the universal fibroblast populations found in other tissues, as revealed by comparative DEG analyses (*61*). While these efforts have already begun in other tissues (e.g., gut) (*62*), future work should focus on establishing a standardized nomenclature for tissue-resident fibroblasts in the pancreas. This would provide a unified framework to advance our understanding of fibroblast roles in pancreatic homeostasis and disease.

Intraorgan mesenchymal heterogeneity is often manifested as spatial heterogeneity, whereby mesenchymal subtypes occupy different positions relative to the epithelium and exert specialized functions. Here, we resolved this spatial organization in the developing pancreas. Comparable findings in the mammary gland (*63*), lung (*64*), and gut (*62*) indicate that this spatial heterogeneity may represent a recurring principle among mesenchymal subtypes across diverse organ systems. We are just at the beginning of our understanding of cell spatial organization and niche formation during organogenesis, with spatial transcriptomics and more recent multimodal approaches providing powerful insights. The application of spatial omics to the pancreas, particularly in the context of development, has remained limited compared to other tissues. A few prior analyses have been performed on human pancreas, in neonatal and adult tissue (*65*) and fetal tissue (12 to 20 PCW) (*66*). However, both these studies relied on the Visium technology, which lacks resolution at the single-cell scale and spatial accuracy, making it suboptimal for defining cell-cell interaction and local niche architecture in embryonic tissue. Our dataset represents the first spatial transcriptomics generated on the mouse pancreas. Unlike the previous studies, using the image-based approach HybISS, we were able to visualize the expression of selected genes within intact tissue samples at single-cell resolution in a multiplex manner. While our targeted approach is constrained by the number of genes measured simultaneously, we show that this limitation can be overcome by predicting gene expression from our reference scRNA-seq onto the cellular-resolution spatial dataset, when needed.

Combining segmentation-free and segmentation-based approaches, we mapped different pancreatic cell types and uncovered tissue niches as epithelial-mesenchymal units. In particular, we focused on disentangling the proximity effect of heterogeneous mesenchymal cell populations in different regions of the tissue. For instance, we found that the M-II subtype is a source of a COLVI-rich ECM, and we demonstrated its proendocrine activity, a feature conserved in both mouse and human. COLVI has been previously identified as a major component of the islet-exocrine interface in the adult pancreas (*40*, *67*); here, we shed light on its role in the embryonic pancreas and, specifically, in endocrine differentiation. Typically, COLVI not only functions via direct engagement of cell surface receptors, such as integrins, but also has the means to sequester cell signaling ligands, such as platelet-derived growth factor, and to regulate the mechanical properties of the cell microenvironment (*68*). Additional studies will be needed to provide a better understanding of the mode of action of COLVI and its biological effects in the pancreas. Ultimately, a broader investigation into cell spatial organization, cell-cell interaction effects, and their mediators will be critical and may lead to previously unknown therapeutic strategies, improving the engineering of complex multicellular tissues for human pancreatic β cells.

Last, our study defined the contribution of embryonic mesenchyme to the adult pancreatic stroma, underscoring the possibility that changes in the mesenchyme composition or niche spatial organization during fetal life might play a role in pancreatic disease. Expanding systematic spatial investigations will be critical to assess whether these deleterious alterations of the endocrine niche in embryonic life could contribute to diabetes pathogenesis.

## MATERIALS AND METHODS

### Mouse strains

All procedures relating to animal care and treatment conformed to the Institutional Animal Care and Research Advisory Committee and local authorities (PPL PP6073640, Home Office, UK). All mouse embryos were used without sex identification (mixed sexes). The following mouse strains were used in this study: Nkx2.5^tm2(cre)Rph^ (*69*), Nkx3.2^tm1(cre)Wez^ (*70*), Tg[Prox1–enhanced GFP (EGFP)] (*28*), and B6.129(Cg)-Gt(ROSA)26Sor^tm4(ACTBtdTomato,-EGFP)Luo^/J (*71*). All mice were bred on a C57BL/6J genetic background. Mice were housed in a specific pathogen–free facility in individually ventilated cages. Room temperature was maintained at 22° ± 1°C with 30 to 70% humidity, and lighting followed a 12-hour light/dark cycle. Food and water were provided ad libitum, and none of the mice had been involved in previous procedures before the study. For timed mating, male and female mice were placed into a breeding cage overnight (ON), and plug check was performed daily. The presence of a vaginal plug in the morning was noted as E0.5.

### Pancreatic explants culture

Dorsal pancreatic buds were microdissected from mouse embryos at E12.5 and cultured on glass-bottom dishes (Matek) precoated with sterile bovine fibronectin (50 mg/ml; Invitrogen) in basal medium Eagle (BME) (Sigma-Aldrich) supplemented with 10% fetal bovine serum (FBS; Invitrogen), 1% glutamine, and 1% penicillin-streptomycin (Invitrogen) (*38*)*.* The day of plating is referred to as day 0. Explants were cultured in a tissue incubator (37°C and 5% CO_2_), and culture medium was changed daily. After 24 hours, BME culture medium was supplemented with BOX5 (50 mg/ml; Sigma-Aldrich) or soluble COLVI (10 mg/ml; Abcam). For matrix experiments, at day 2 of culture, the medium was removed, and the explants were overlaid with the matrix solutions, which were allowed to polymerize for 30 min at 37°C. BME medium was then added to the plate, and the explants were cultured for three additional days. Collagen hydrogel solutions were prepared by mixing COLI (Serva) with or without COLVI (Abcam), BME medium, and 10× phosphate-buffered saline (PBS) and neutralized using NaOH. Matrigel mixture was prepared by mixing BME medium and Matrigel (Corning) in a 1:1 proportion. At the end of the treatment, the explants were briefly washed with PBS, fixed for 20 min at 4°C in 4% paraformaldehyde (PFA), and processed for whole-mount IF, as previously described (*72*).

### Immunohistochemistry and RNAscope

Mouse embryos and pancreata were fixed in 4% PFA at 4°C from 2 hours to ON. Human embryonic and fetal tissue was provided by the joint Medical Research Council/Wellcome Trust (grant nos. MR/X008304/1 and 226202/Z/22/Z) Human Developmental Biology Resource (HDBR; http://hdbr.org) with appropriate maternal written consent and approval from the Newcastle and North Tyneside NHS Health Authority Joint Ethics Committee (23/NE/0135) and London Fulham Research Ethics Committee (23/LO/0312). The HDBR is regulated by the UK Human Tissue Authority (HTA; www.hta.gov.uk) and operates in accordance with the relevant HTA Codes of Practice. Human pancreatic tissue samples (gender not established) were fixed ON in 4% PFA and then processed for cryosectioning, immunostaining, and imaging at King’s College London. All work was undertaken in approval of the HDBR Steering Committee to the Spagnoli laboratory at King’s College London, UK (license no. 200523).

For cryosectioning, samples were equilibrated in 20% sucrose solution, embedded in O.C.T. compound (Tissue-Tek, Sakura) and sectioned at 10 μm in thickness. For immunostaining, sections were incubated in trichostatin A (PerkinElmer) blocking buffer for 1 hour at room temperature. If necessary, then antigen retrieval was performed by boiling slides for 20 min in citrate buffer (Dako). Sections were incubated in primary antibody solution (3% horse serum and 3% bovine serum albumin in PBS) at the appropriate dilution (see table S8) ON at 4°C. Hoechst 33342 nuclear counterstaining was used at a concentration of 250 ng/ml. For RNAscope in situ hybridization, cryosections were processed with the RNAscope Multiplex Fluorescent Reagent Kit v2 (Advanced Cell Diagnostics) according to the manufacturer’s instructions. Protease Plus (diluted 1:5) was applied to permeabilize samples for 10 min. Probes (see table S7) were used in combination with Opal 650 and Opal 570 fluorophores. Images were acquired with Zeiss AxioObserver, Zeiss Discovery and Zeiss LSM 700 laser scanning confocal microscopes.

### Tissue clarification and light-sheet microscopy

Whole-mount IF staining on pancreatic tissue was performed as previously described (*73*). After whole-mount staining, tissue clarification was performed using CUBIC1 [25% (w/v) urea, 25% (w/v) *N*,*N*,*N*′,*N*′-tetrakis(2-hydroxypropyl) ethylenediamine, and 15% (w/v) Triton X-100 in distilled H_2_O (dH_2_O)] and CUBIC2 [50% (w/v) sucrose, 25% (w/v) urea, 10% (w/v) 2,20,20′-nitrilotriethanol, and 0.1% (v/v) Triton X-100 in dH_2_O] solutions. After clarification, samples were glued to a 1-ml syringe support holder using all-purpose super glue. The syringe was then inserted into the syringe holder provided with the Zeiss Z1 light-sheet microscope. Samples were then imaged with the Zeiss Z1 light-sheet microscope using 20× acquisition and 10× illumination lenses.

### Library preparation and RNA-seq

Pancreatic tissue from E12.5 Tg(Prox1-EGFP), *Nkx2.5*-Cre;*R26*mTmG, and *Nkx3.2*-Cre;*R26*mTmG embryos was manually dissected in diethyl pyrocarbonate (DEPC)–PBS, individually digested in collagenase for 10 min at 37°C, and processed for FACS sorting, as previously done in (*73*). Tissue digestion was blocked by adding Dulbecco’s modified Eagle’s medium with 10% FBS, and cells were spun at 300*g* for 3 min at 4°C, resuspended in DEPC-PBS, and filtered through FACS tubes with Cell Strainer Cap (BD, 352235) for immediate FACS sorting (BD FACSAria II or III). Single cells were sorted into 96-well plates in lysis buffer [0.2% Triton X-100 and RNasin (2 U/μl) in nuclease-free water]. Library preparation and transcriptome sequencing were performed by the Genomic sequencing facility at Department of Biosystems Science and Engineering (Basel), according to the Smart-seq2 protocol (*73*). Sequences were obtained from E12.5 GFP^+^ cells from Nkx2.5-Cre;R26mTmG (192 cells), Nkx3.2-Cre;R26mTmG (192 cells), and Tg(Prox1-EGFP) (193 cells). cDNA profiles were checked on the Fragment Analyzer (AATI), and their concentration was determined using the Quant-iT PicoGreen dsDNA Assay Kit. Libraries were pooled and sequenced SR75 on an Illumina NextSeq 500 system (75 cycles of High Output v2.5 kit).

### scRNA-seq processing

Reads were uniquely aligned to the mouse reference genome (GRCm38) using Kallisto pseudomode (v.0.46.1). The gene expression matrices were generated by converting transcript level counts to gene-level counts using tximport (v.1.18.0).

Downstream analyses were completed with Seurat (version 4.4.0) (*74*). Quality control was performed to remove genes expressed by less than three cells and exclude cells expressing fewer than 1000 genes, less than 100,000 gene counts, or a percentage of counts derived from the mitochondrial genome higher than 30%. Data were log normalized, and cell cycle differences were regressed using the ScaleData function. We used principal components analysis (PCA) for dimension reduction and unsupervised clustering of the data with the FindNeighbors() and FindClusters() functions. Meaningful PCAs were selected using the Jackstraw function and used for the FindNeighbors() function to construct a shared nearest-neighbor graph of all the data. Then, the cells were clustered using the function FindClusters() with a shared nearest-neighbor modularity optimization–based clustering algorithm with a resolution of 1.9. To visualize the data, we used UMAP. Clusters were lastly interrogated and manually curated on the basis of their DEGs.

E12.5, E14.5, and E17.5 datasets (GSE101099) (*17*) were obtained from the Gene Expression Omnibus (GEO) database. Quality control was performed using values specified by the authors. To integrate it with our Smart-seq2 dataset, each separate batch was preprocessed independently using the SCTransform() function (*75*) with 3000 features and regression of differences in cell cycle state among cells. Integration was then performed using the Seurat integration workflow. Downstream processing was performed as previously specified. The first 20 PCAs were used for the FindNeighbors() functions, and a resolution of 1 was used to find clusters. For the analysis of M-VSM cells, PCA were recomputed after subclustering. The first 20 PCAs were used for the FindNeighbors() function, and a resolution of 0.5 was used to find subclusters.

Adult scRNA-seq datasets were obtained from published datasets GEO (GSE125588 and GSE176063) (*3*, *48*) and ArrayExpress database (E-MTAB-8483) (*47*). Quality control was performed using values specified by the authors. For integration, each separate batch was preprocessed independently using the SCTransform() function (*75*) with 3000 features and regression of differences in cell cycle state among cells. Integration was then performed using Seurat integration as previously specified. The first 30 PCAs were used for the FindNeighbors() functions, and a resolution of 0.7 was used to find clusters. For adult and embryonic dataset integration, adult and embryonic mesenchymal cells were integrated using the Harmony package (*76*). The first 50 PCAs were used for the FindNeighbors() functions, and a resolution of 0.5 was used to find clusters.

Human scRNA-seq data were obtained from the OMIX database (OMIX001616) (*27*). Quality control was performed using values specified by the authors. After subsetting mesenchymal cells from each batch, integration was performed using the Seurat workflow as previously specified. For human-mouse integration, mouse orthologs were first mapped to human genes using the Python package mousipy (v.0.1.6). Next, human and mouse datasets were integrated using MultiMAP (*77*) using separately precalculated principal with 0.7 and 0.3 strengths, respectively. DEGs were found using the Seurat’s FindAllMarkers() function.

Cluster comparison between our embryonic integrated dataset and Scavuzzo *et al.* (*26*) dataset was performed by reprocessing their E14.5 Drop-Seq dataset (GSE100622) reusing the same steps used to process the integrated embryonic dataset, and clustering was performed so that the resulting clusters fitted those described in the original publication. Then, FindTransferAnchors() and TransferData() Seurat functions were used to transfer labels from E14.5 subset cells from the integrated dataset to the Scavuzzo *et al.* (*26*) dataset. To generate signatures of Scavuzzo *et al.* (*26*) clusters, we used the list of DEGs obtained from the original publication. For each cluster, the top 10 genes expressed in each cluster were used to generate signatures using the AddModuleScore() Seurat function in the embryonic dataset used throughout this study.

### L:R interaction analysis

CellChat (v.1.6.1) (*31*) was used to investigate cellular interactions based on our Smart-seq2 dataset. First, we used the CellChatDB L:R interaction database and then the CellChat pipeline to compute the interaction number and strength between the different cell types, as well as the incoming and the outgoing signaling patterns and the specific L:R interactions using default parameters.

### Matrisome analysis

Matricom (v 1.0) (*41*) was used to investigate communication between ECM components and different cell populations on our Smart-seq2 dataset. Interactions were obtained using the web interface (https://matrinet.shinyapps.io/matricom) and default parameters.

### Diffusion analysis

To perform diffusion analysis on our Smart-seq2 dataset, we used Scanpy (v.1.9.0) (*78*). First, we excluded epithelial, mesothelial, and proliferating mesenchymal cells. Then, we generated a normalized, log-transformed cell-by-gene matrix of 2000 variable genes, which we used to generate diffusion maps using the Scanpy tl.diffmap() function. Meaningful DCs were used to generate a diffusion plot, which we subsequently used to visualize cell identities, gene expression, and gene module expression. To investigate gene expression trends associated with underlying transcriptional heterogeneity, we used the pyGAM package (v. 0.8.0). Specifically, we used the 1000 most variable genes and fit their gene expression along DC1. Significantly associated genes were defined as those having *P* < 0.01. Gene module analysis was performed with the GSEApy package (v.1.1.4) to retrieve gene signatures from BioCarta (namely, “Signaling by Hippo,” “ERK MAPK Targets,” and “Signaling by WNT”). The Scanpy tl. tl.score_genes() function was then applied to generate signature scores.

For the FGF9 KO analysis, we retrieved up-regulated and down-regulated gene signatures from (*32*). The Scanpy tl.score_genes() function was then applied to generate signature scores that were subsequently plotted in the diffusion plot.

### PAGA and Slingshot trajectory analysis

To calculate and plot the simplified graph embedding of the mesenchymal clusters, we used Scanpy tl.paga() and pl.paga() functions after subsetting mesenchymal clusters of the Smart-seq2 dataset. For the trajectory analysis in the embryonic and adult integrated mesenchyme dataset, we used Slingshot (v.2.8.0) (*79*). On the basis of the PAGA (*50*) graph of the Smart-seq2 dataset and the levels of *Nkx2.5* expression (fig. S10A), the M-SpP was set up as the root for the calculation of lineages and trajectories.

### HyISS for spatially resolved transcriptomics

E12.5, E14.5, and E17.5 embryonic pancreata were dissected and fixed in 4% PFA ON. Samples were equilibrated in 20% sucrose solution and embedded in O.C.T. compound (Sakura). Ten-micrometer-thick cryosections were stored at −80°C. On the starting day of the experiment, sections were incubated at 60°C for 1 hour before postfixation with 4% PFA for 5 min. After washing, sections were serially dehydrated in 50, 70, and 100% ethanol, subjected to antigen retrieval in citrate buffer, and then hybridized with the gene panels at 37°C ON. Ligation and following steps were performed using the High Sensitivity Library Preparation Kit from Cartana AB (10x Genomics) as described (*14*). Incubations were performed in SecureSeal chambers (Grace Biolabs, Bend, USA). SlowFade Antifade Mountant (Thermo Fisher Scientific, Waltham, MA, USA) was used for optimal handling and imaging of tissue sections. After quality control, samples were processed by Cartana AB (10x Genomics) for in situ barcode sequencing, imaging, and data processing. This generated an output consisting of DAPI images of tissue sections and CSV files, containing gene identity and position of identified RNA spots. To generate gene expression tissue plots, we used MATLAB. For downstream analysis, we manually generated a mask around the pancreas to filter out cells and reads from other tissues. E12.5 and E14.5 datasets were analyzed by segmentation-free and segmentation-based computational frameworks, while the E17.5 dataset was used only for qualitative analysis due to the limited number of sections analyzed and low detection rate.

### Cell segmentation–free approach (SSAM)

Segmentation-free analysis of the HybISS data was performed following the SSAM pipeline (v.1.0.2) (*22*). Gene reads coordinates were first transformed from pixels into micrometers (0.32 μm per pixel) and then used to generate mRNA density maps through a kernel density estimation (Gaussian kernel and a bandwidth of 2.5). Gene density maps were then combined to identify local maxima using a total gene expression threshold of 0, a per-gene expression threshold of 0, and a search size of 3. Local maxima were then used to calculate the variance stabilization parameters with the SCTransform package (v0.3.4) (*75*), which were subsequently used for gene expression normalization. For cell type identification, we used the integrated dataset containing E12.5, E14.5, and E17.5 cells as the reference dataset. The raw gene counts were normalized using SCTransform and the average gene expression per cell type was calculated. Local maxima vectors were mapped to the cell type clusters with the most similar gene expression in the scRNA-seq reference dataset, using a correlation minimum threshold of 0 and a threshold of vector normalization of 0.025. The obtained tissue maps of each section were then analyzed independently to build tissue domains. The combined list of tissue domains from all samples was then clustered and visualized using *k*-means clustering, PCA, and UMAP. Tissue domains were lastly manually annotated on the basis of the most abundant cell types in each domain.

### Cell segmentation–based approach

For the segmentation-based approach, we first used Cellpose 2.0 (*24*) to segment cells based on DAPI staining. Next, we assigned reads to each cell using probabilistic cell typing for in situ sequencing (*25*)*.* This generated a cell-by-gene matrix, which was then used as input for Tangram (*23*) to both annotate cell type and impute gene expression based on the integrated scRNA-seq dataset. The resulting tissue maps were then combined and used to build neighborhood enrichment plots using Squidpy (*80*)*.* Squidpy (v.1.1.4) was used to compute a spatial graph with the gr.spatial_neighbors() function and a window of 10 neighbors. We then used this graph to compute neighborhood enrichment scores.

### Cell lines and cell culture

Human iPSC line HMGUi001-A2 (sex, female) (*81*) was provided by H. Lickert (Helmholtz, Munich) and authenticated by karyotyping. Human iPSCs were maintained on Geltrex-coated (Invitrogen) plates in E8 medium. The medium was changed daily, and cells were passaged every 3 to 4 days as cell clumps or single cells using 0.5 mM EDTA (Invitrogen) or Accutase (Invitrogen), respectively. The medium was supplemented with rho-associated protein kinase (ROCK) inhibitor Y-27632 (10 mM; Sigma-Aldrich) when iPSCs were thawed or passaged as single cells.

### Differentiation of pluripotent iPSCs into pancreatic β-like cells

Human iPSC differentiation was carried out following a protocol adapted from published ones (*82*, *83*) (table S9). Briefly, iPSCs were dissociated using Accutase and seeded at a density of 2.8 × 10^6^ cells per well in six-well plates (Corning) coated with Geltrex (Invitrogen) in E8 medium supplemented with ROCK inhibitor (10 mM). Cells were differentiated into pancreatic progenitors as previously described (*82*, *83*). At day 11 of the protocol, pancreatic progenitors were dissociated using Accutase and seeded to microwells (24-well AggreWell 400 microwell plates, STEMCELL Technologies) at a density of 1000 cells per microwell. Cells were maintained in stage 4 medium in AggreWell for 3 days. On the first day of stage 5, endocrine progenitors were transferred from AggreWell to ultralow attachment plates (CELLSTAR) and placed on an orbital shaker for suspension culture at 100 rpm. At day 4 of stage 5, three-dimensional (3D) cell clusters were collected and resuspended in Matrigel or collagen hydrogel mixtures. The matrix was allowed to polymerize for 30 min at 37°C. Subsequently, the medium was added to the plate, and clusters were cultured for an additional 5 days in stage 6 medium. Collagen hydrogels were prepared as described for pancreatic explants.

### Quantifications, statistics, and reproducibility

Cell numbers and immunostaining intensities were quantified on sections and explants using QuPath (*84*). For cell quantification in embryonic samples, the entire pancreas was serially sectioned (10 μm in thickness). For cell counting, positive cells (matching a Hoechst^+^ nucleus) were manually counted or using the QuPath positive cell detection function. The relative area occupied by cells per pancreas was obtained by normalizing the counted immunopositive cells to the total E-cadherin^+^ or Pancreatic And Duodenal Homeobox 1 (PDX1)^+^ epithelium area.

For cell quantification in whole-mount pancreatic explants, immunopositive cells were counted in three focal planes of a Z-stack (top, center, and bottom) and normalized to the average epithelial area (X.Y), multiplied by the number of focal planes analyzed in the explant (Z). Analysis of mesenchymal cell positioning along the gut-splenic axis was performed by measuring IF intensities in nonepithelial cells (E-cadherin^−^ or PDX1^−^) and their distance to the spleen using QuPath. IF intensities were corrected by linear normalization within each embryo to achieve a uniform dynamic range and improve comparability between embryos. To show the curves fitting the data, we used GraphPad function spline curves with five knots. Quantification of *Wnt5a* expression and fluorescent intensity (LEF1, pJNK, and pJUN) in embryonic tissue sections and explants was performed using QuPath. Confocal images used for the analysis were acquired with the same laser settings for both conditions and were not altered before quantification. In adult samples, quantifications were performed across five to seven independent fields of view in each of three different sections per adult pancreas. For cell quantification in iPSC-differentiated clusters, we used the “Spots” function in Imaris software.

Experiments were repeated a minimum of three times; one representative field of view is shown for each staining. All results are expressed as means ± SD or means ± SEM, as indicated. Sample sizes of at least *n* = 3 were used for statistical analyses except where indicated.

Data representation and statistical analysis were performed using GraphPad Prism (version 9) and Excel (Microsoft). Statistical significance was determined as indicated in figure legends using unpaired two-tailed Student’s *t* test, Mann-Whitney test, or analysis of variance (ANOVA) test for more than two groups. Statistical analysis was conducted on all collected samples and data. No statistical method was used to predetermine the sample size. No data were excluded from the analyses. The experiments were not randomized. Investigators were not blinded to allocation during experiments and outcome assessment.
