# Supplementary material for: Spatially organized cellular communities shape functional tissue architecture in the pancreas
Source: Sci Adv. 2025 Nov 12;11(46):eadx5791. doi: 10.1126/sciadv.adx5791 (PMC12609055; doi:10.1126/sciadv.adx5791)
Supplement: Supplementary file 1 — Figs. S1 to S10 Legends for tables S1 to S9 [file sciadv.adx5791_sm.pdf]

Science Advances
AAAS

Supplementary Materials for

Spatially organized cellular communities shape functional tissue architecture in the pancreas

Alejo Torres-Cano *et al.*

Corresponding author: Francesca M. Spagnoli, francesca.spagnoli@kcl.ac.uk

Sci. Adv. **11**, eadx5791 (2025)
DOI: 10.1126/sciadv.adx5791

The PDF file includes:

Figs. S1 to S10
Legends for tables S1 to S9

Other Supplementary Material for this manuscript includes the following:

Tables S1 to S9

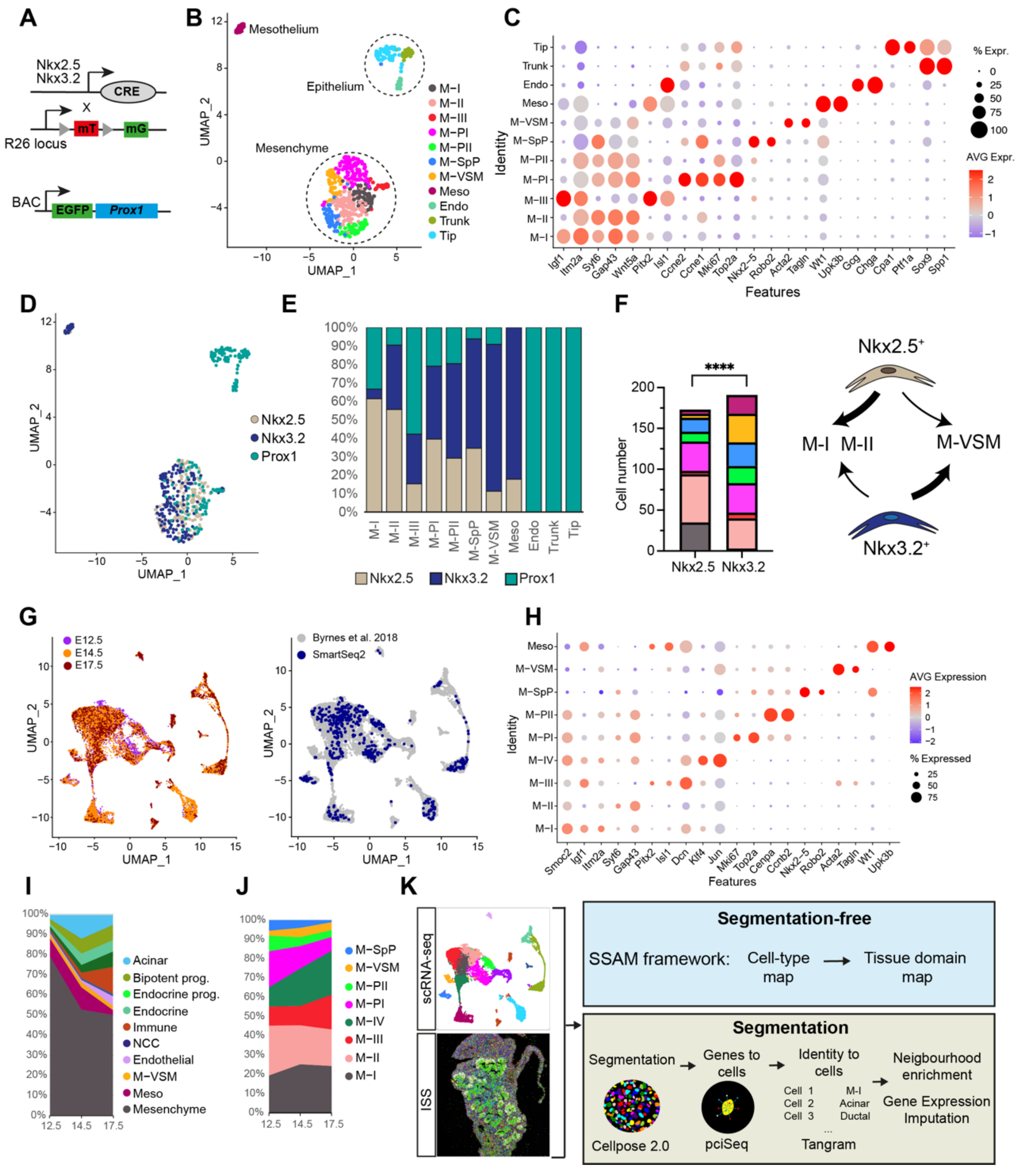

Fig. S1. ScRNAseq analysis of Nkx2.5- and Nkx3.2-descendant mesenchymal cell types in the pancreas. (**A**) Smart-seq2 sequencing of GFP-positive cells FACS-sorted from E12.5 dorsal pancreas of *Nkx2.5*-Cre;*R26*mTmG, *Nkx3.2*-Cre;*R26*mTmG and Prox1-GFP BAC transgenic (Tg) mouse embryos. *Nkx2.5*-Cre and *Nkx3.2*-Cre recombined in specific subpopulations of the dorsal pancreas mesenchyme (*16,19*), whereas *Prox1* was expressed mainly in the pancreatic epithelium (*28*). (**B**) UMAP plot showing clustering of Smart-seq2 scRNAseq profiles of embryonic pancreatic tissue at E12.5. After batch correction and clustering we identified 11 populations, including 3 epithelial (E) progenitor clusters (Tip, Trunk and Endocrine cells), 1 cluster expressing hallmark mesothelial genes (Meso), like *Upk3b* and *Wt1*, and 7 mesenchymal (M)

clusters. Clusters are colour coded to indicate their annotated cell type. (**C**) Dot plot showing the expression level for selected marker genes for each cluster from (B). Proliferative clusters (M-PI and M-PII) are enriched in genes associated with cell cycle progression (*Mki67*, *Top2a*); the M-VSM cluster is enriched in genes expressed by mural cells (*Acta2*, *Tagln*); the spleno-pancreatic mesenchyme (M-SpP) cluster expresses high levels of transcription factors, such as *Nkx2.5* and *Tlx1*; M-I is characterized by high expression of *Itm2a* and *Igf1;* M-II is enriched in genes coding for collagens (*Col-6*), non-canonical Wnt pathway (*Wnt5a*) and axon guidance (*Slit2*); M-III displays high levels of expression of *Pitx2* and *Isl1*, previously reported for a role in early stage pancreatic mesenchyme *(17,18,26)* (table S1). Colour bar indicates the linearly scaled mean of expression level. (**D**) UMAP visualization of Smart-seq2 scRNAseq with cells coloured by the 3 Tg lines of origin. (**E**) Plot showing distribution of Tg cells across clusters (shown as %). Columns represent clusters; colours indicate the Tg line of origin. (**F**) Stacked bar plot showing the number of Nkx2.5- and Nkx3.2-mesenchymal cells contributing to each cluster ($\chi 2$, p<0,0001). Nkx2.5^{+} progenitors preferentially give rise to M-I and M-II clusters, while Nkx3.2^{+} progenitors to M-VSM cells. (**G**) UMAP plot of publicly available scRNAseq datasets *(17)* and Smart-seq2 of transgenic pancreatic rudiments (see Fig. 1B) after integration, coloured by embryonic stage (left panel) or by datasets (right panel). After batch correction and clustering, 17 cell populations were identified. These included known epithelial subtypes in the pancreas (acinar, bipotent endocrine-duct, endocrine progenitors and endocrine cells), neural crest-derived cells (*Phox2b*$^{+}$), immune cells (*Cd74*$^{+}$), endothelial cells (*Pecam1*$^{+}$), blood cells (*Alas2*$^{+}$), mesothelial (*Upk3b*, *Wt1*) and eight mesenchymal clusters, including M-I to M-III subtypes, two proliferative (M-PI and M-PII) clusters, one M-IV cluster (*Klf4*, *Jun*, *Fos*), one M-VSM cluster enriched in genes expressed by vascular mural cells (*Acta2*, *Tagln)* as well as pericytes, and one cluster expressing high levels of M-SpP genes. (**H**) Dot plot showing the expression level for selected marker genes for each mesenchymal (M) and mesothelial (Meso) clusters from the integrated scRNAseq dataset (as in Fig. 1B). Colour bar indicates the linearly scaled mean of expression level (table S2). (**I**) Proportion of cell clusters per developmental stage. (**J**) Proportion of mesenchymal cluster subtypes per developmental stage. (**K**) Schematics of the spatial transcriptomic analysis framework. HybISS and scRNAseq datasets were combined and analysed following segmentation-free and segmentation-based complementary approaches (See Materials & Methods section).

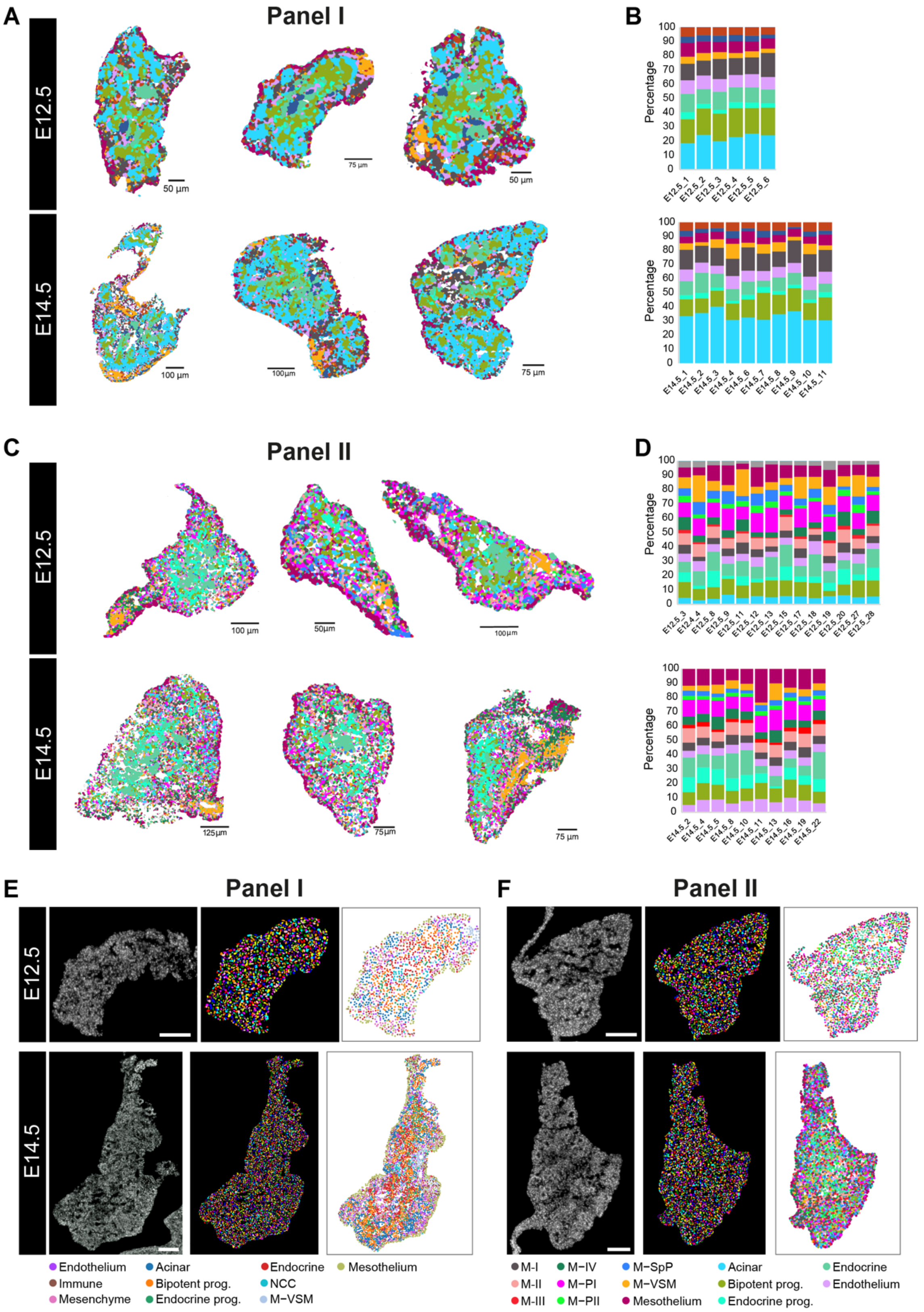

Fig. S2. Segmentation-free and segmentation-based analyses of the HybISS datasets. (**A**) Representative SSAM-annotated cell maps of the HybISS Panel I experiment on E12.5 and E14.5 pancreatic tissue sections. (**B**) Cell type proportions per sample from E12.5 and E14.5 HybISS Panel I experiment. Colours represent single-cell clusters as

shown below (E). (**C**) Representative SSAM-annotated maps of the HybISS Panel II experiments on E12.5 and E14.5 pancreatic tissue sections. (**D**) Cell type proportions per sample from E12.5 and E14.5 HybISS Panel II experiment. Colours represent single-cell clusters as shown below in (F). (**E,F**). Representative nuclear staining of E12.5 and E14.5 sections from Panel I (E) and II (F) (left). Nuclear segmentation staining generated using Cellpose 2.0 (middle); segmented nuclei are randomly coloured. Real position of annotated HybISS cells (right). Colours represent cell clusters as in Fig. 1A. Scale bar, 100μm.

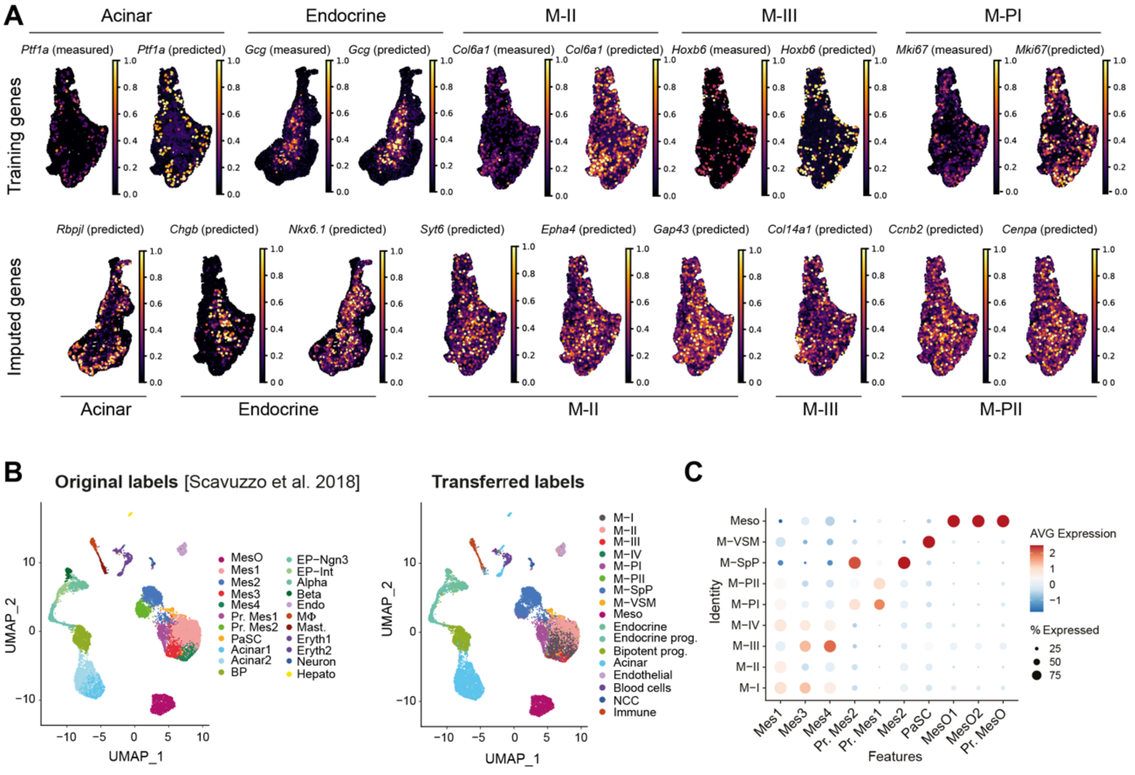

D

Cell type annotation from(26)	Cell type annotation from this study	Markers
Mes1	M-II	Gap43, Slit2, Igf1-
	M-I	Igf1, Smoc2
Mes3/4	M-III	Sfrp2, Dcn
	M-IV	Jun, Klf4
Pr. Mes1	M-PI	Mki67, Top2a
	M-PII	Cenpa, Ccnb2
Mes2/Pr. Mes2	M-SpP	Nkx2.5, Itga8
PaSC	M-VSM	Acta2, Tagln
MesO	Meso	UpK3b, Wt1

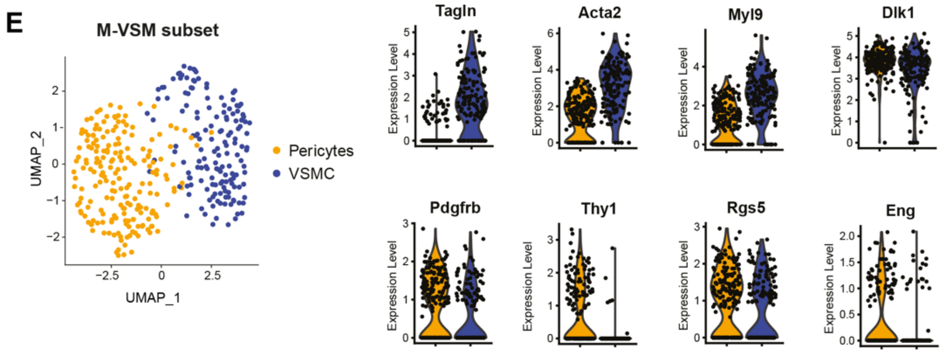

Fig S3. Mapping sc-profile onto spatial profile. (**A**) Measured (reference) and predicted (inferred) gene expression of selected cell-type markers based on scRNAseq imputation of gene expression profiles in E14.5 sections from Panel I (*Gcg, Rbpjl, Nkx6.1*) and Panel II (*Ptf1a, Col6a1, Hoxb6, Mki67, Syt6, Epha4, Gap43, Col14a1, Ccnb2*). (**B**) UMAP plots showing clustering of scRNAseq profiles of embryonic pancreatic tissue at E14.5 obtained from Scavuzzo et al. *(26)* and labelled as in the original publication (left) or with

labels transferred from our integrated dataset shown in Fig. 1A. (**C**) Dot plot showing the expression level of signatures from Scavuzzo et al. *(26)* clusters in mesenchymal clusters from this study. Colour bar indicates the linearly scaled mean of expression level. (**D**) Summary of mesenchymal cell cluster comparison and shared markers across indicated studies. Substantial overlap in molecular signatures is present across datasets, in particular regarding the mesothelium, proliferative mesenchyme, M-VSM. While similar consistency can also be noted for other mesenchymal clusters, they were labelled differently in the other dataset *(26)*. (**E**) UMAP plot of M-VSM vascular mesenchyme cells distinguishing two individual subpopulations. Sub-clustering of M-VSM revealed that it is composed of vascular smooth muscle cells (VSMC), characterized by high expression of *Tagln, Acta2 and Myl9*, and pericytes showing high expression of *Thy1, Rgs5, Eng,* as previously reported *(8)*. Right: Violin plots showing the expression of selected markers in pericytes and VSMC populations.

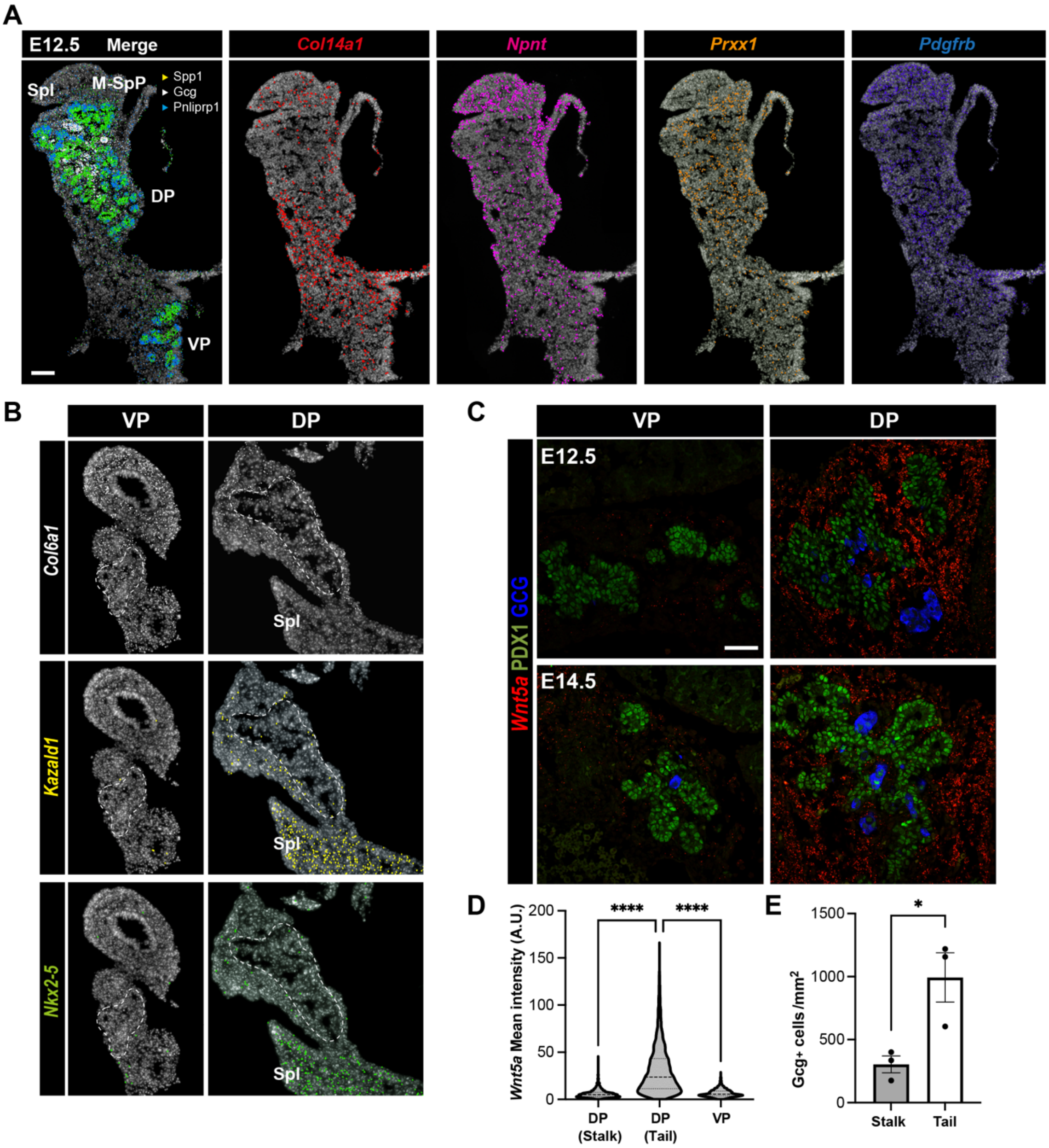

Fig. S4. Dorsal and ventral pancreas show differences in their mesenchymal composition. (**A**) Representative HybISS images showing spatial distribution of indicated pancreatic reference genes (left panel) and mesenchymal marker genes (right panels) at E12.5. DP, dorsal pancreas; M-SpP, spleno-pancreatic mesenchyme; Spl, spleen; VP, ventral pancreas. Scale bar, 100µm. (**B**) Representative HybISS images showing spatial distribution of selected mesenchymal genes in VP (left) and DP (right) sections at E12.5. White dotted lines demarcate the pancreatic epithelium. (**C**) Representative confocal microscopy IF images of E12.5 and E14.5 DP and VP cryosections stained for indicated markers. Scale bar, 50µm. (**D**) Quantification of *Wnt5a mRNA* transcript levels on RNAScope-labelled DP at stalk and tail regions and VP tissues. AU, arbitrary units. n=3 embryos. ****$p < 0.0001$; One-way Anova test. (**E**)

Quantification of glucagon (Gcg)$^+$ cells in IF images. Cell counts were normalized to the average pancreatic epithelium area (mm2) and shown per area in stalk and tail regions of DP tissue. n=3 embryos. Error bars represent ± SEM. *$p < 0.05$; two-tailed unpaired t test.

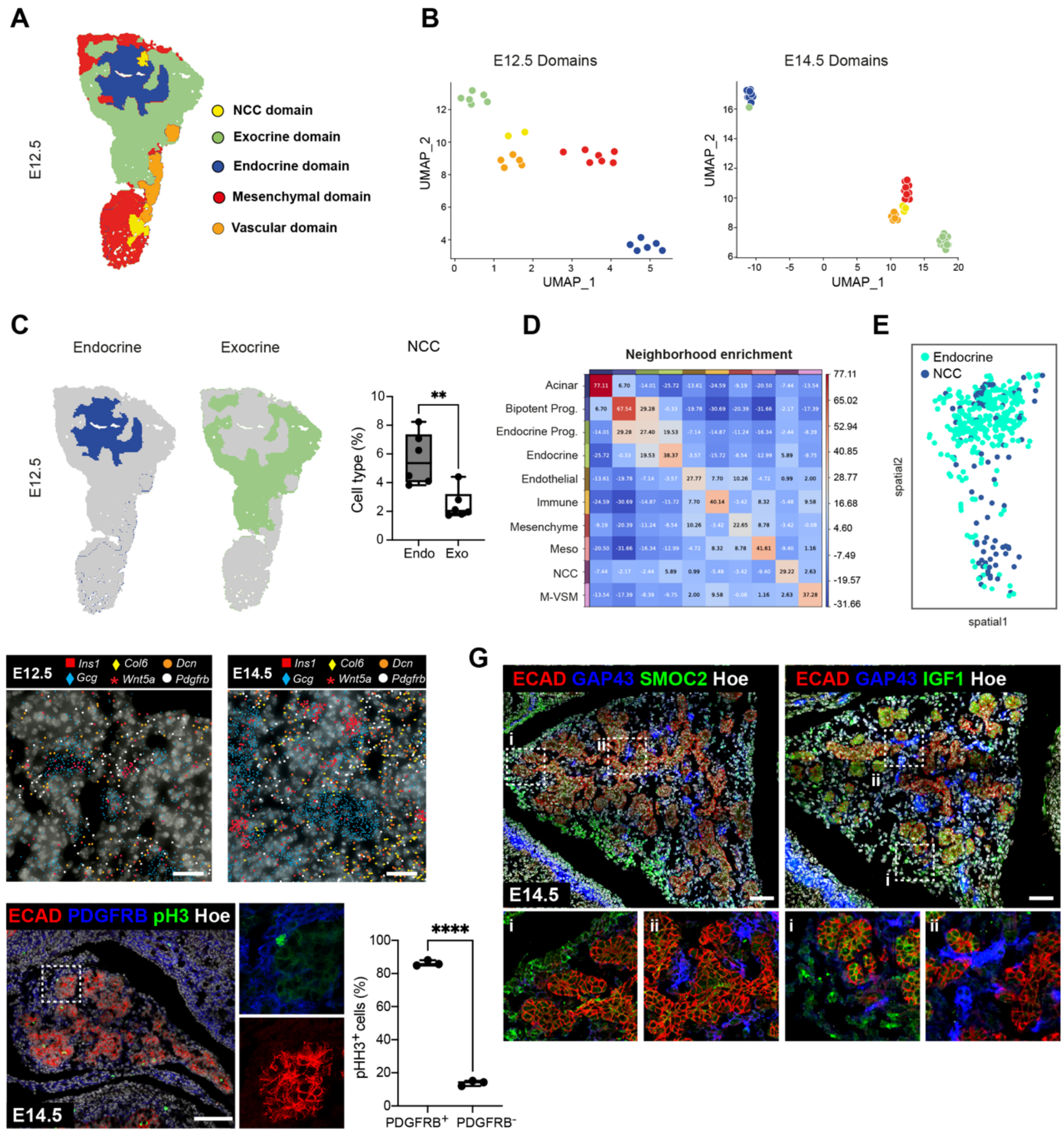

Fig. S5. SSAM-based tissue domain annotation on images from Panel I dataset. (**A**) Representative SSAM-annotated tissue domain map from HybISS Panel I dataset at E12.5. (**B**) UMAP plots show annotated tissue domains at E12.5 and E14.5, identified by SSAM performed on all tissue images from Panel I dataset. Each point represents a predicted tissue domain from an individual tissue image. A list of tissue domains along with their cell composition is included in table S4. (**C**) Representative endocrine and exocrine tissue domain maps of E12.5 pancreatic tissue (left) and quantification of NCC populations in each domain (right). **$p < 0.01$; two-tailed unpaired t-tests. (**D**) Neighbourhood enrichment score between cell types in E12.5 pancreatic tissue Panel I dataset (shown as mean; $n = 6$ tissue sections). Positive enrichment indicates proximity of a particular cell type to another one. Z-score indicates if a cluster pair is over-

represented or over-depleted in the analysis. (**E**) Spatial distribution of NCC and endocrine cell types identified by HybISS on a E12.5 pancreatic tissue section. This is consistent with a previously reported role of NCC-derived cells in endocrinogenesis *(7)*, highlighting their importance in the endocrine niche. (**F**) Representative HybISS images showing selected genes expressed by endocrine and M-II cells from Panel II at E12.5 and E14.5. Scale bar, 25µm. (**G**) Representative confocal images of E14.5 pancreatic tissue sections stained for E-cadherin (ECAD), the M-II marker, GAP43, with M-I and M-III markers (IGF1, SMOC2). Bottom panels show magnification of boxed regions at the periphery (i) and core (ii) of the organ. Hoechst was used as nuclear counterstain. Scale bar, 100µm. (**H**) Representative confocal image of a pancreatic cryosection stained for E-cadherin (ECAD), the proliferation marker pH3 and pan-mesenchymal marker PDGFRB. Right panels show magnification of boxed region. Hoechst was used as nuclear counterstain. Scale bar, 150µm. Quantification of proliferative cells outside the pancreatic epithelium ($ECAD^-$) is shown on the right. The majority of proliferative non-epithelial cells ($ECAD^-$ $pH3^+$) are $PDGFRB^+$ mesenchymal cells, a small fraction ($PDGFRB^-$) belongs to other microenvironment cell types.

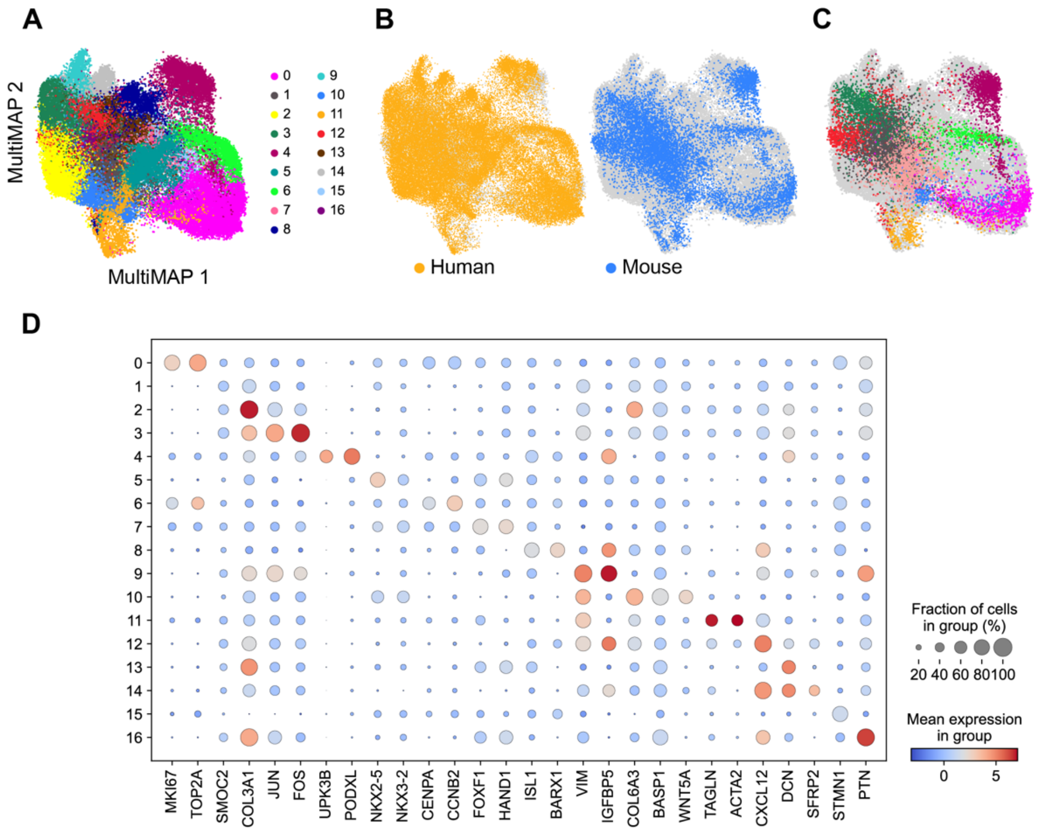

Fig. S6. ScRNAseq comparison between human and mouse mesenchyme cells of the pancreas. (**A-C**) MultiMAP visualization of mesenchymal cells from human *(27)* and mouse foetal pancreas coloured by cell cluster (A), species (B) or by annotated mouse clusters (C). N/A in (C) corresponds to human cells. (**D**) Dot plot showing the expression level of marker genes for each cell cluster from the integrated scRNAseq dataset [as shown in (A)]. Colour bar indicates the linearly scaled mean of expression level.

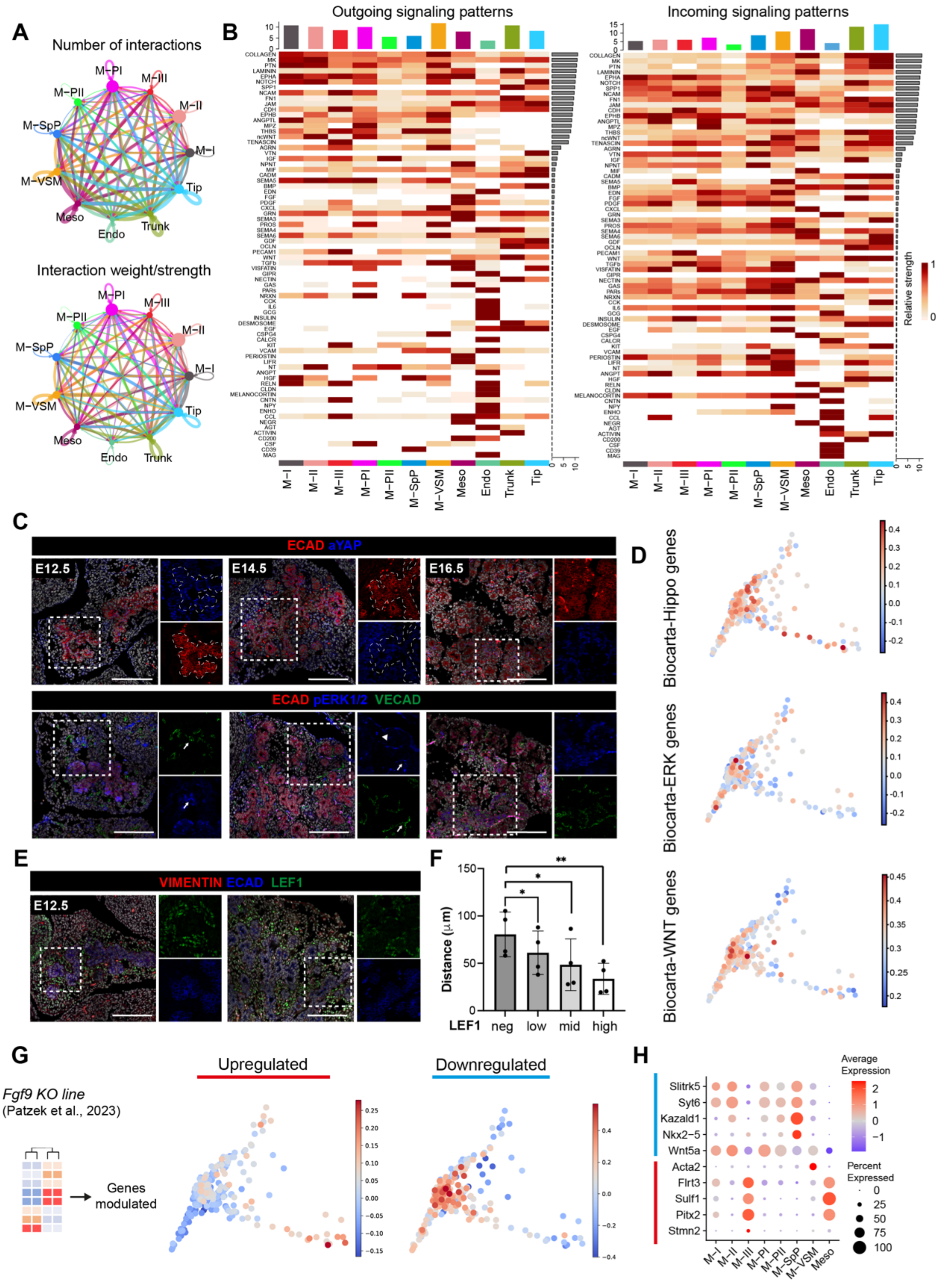

Fig. S7. Cell signalling in the pancreatic mesenchyme. (**A**) Chord diagrams showing the cell-cell communication networks between pancreatic mesenchyme and epithelial cell types based on Cellchat analysis performed on our E12.5 Smart-seq2 dataset (see fig. S1A). (**B**) Heatmaps indicate the signalling strength of outgoing (ligands) and incoming (receptors) signalling pathways in each cell type. Signalling pathways are ordered by overall strength of signalling. (**C**) Representative confocal images of DP cryosections

stained for the indicated signalling molecules. Right panels show boxed regions as single channels. White dotted lines demarcate the pancreatic epithelium. Arrows indicate p-ERK localization cells next to VECAD^{+} endothelium; arrowhead indicates p-ERK localization in mesothelial cells. Hoechst was used as nuclear counterstain. Scale bar, 150µm. (**D**) Diffusion plots of mesenchymal scRNAseq profiles showing average expression levels of normalized and log-transformed levels of gene modules from indicated signaling pathways based on BioCarta gene annotation (see Fig. 2E for M-clusters). (**E**-**F**) IF and quantification analysis of LEF1^{+} cell average distance to pancreatic epithelium (marked by ECAD staining). Scale bar, 150µm. LEF1^{+} cells were preferentially found next to the epithelium, suggesting a Wnt signaling gradient across the pancreatic mesenchyme. Right panels show boxed regions as single channels. Hoechst was used as nuclear counterstain. n=4 embryos. Error bars represent ± s.d.. *$p < 0.05$, **$p < 0.01$; One-way Anova test. (**G**) Gene signatures obtained from bulk RNAseq data of pancreatic tissue, isolated from *Fgf9* knockout (KO) mice *(32)*, were projected into the Smart-seq2 E12.5 mesenchyme dataset diffusion plot. Genes downregulated in *Fgf9* KO were expressed in M-clusters closer to the spleen, whilst genes upregulated in the *Fgf9* KO were expressed in M-clusters closer to the duodenum, as defined by the transcriptional dynamics characterized in Fig. 2. (**H**) Dot plot showing a subset of the genes measured in (G) in E12.5 Smart-seq2 M-clusters. Colour bar indicates the linearly scaled mean of expression level.

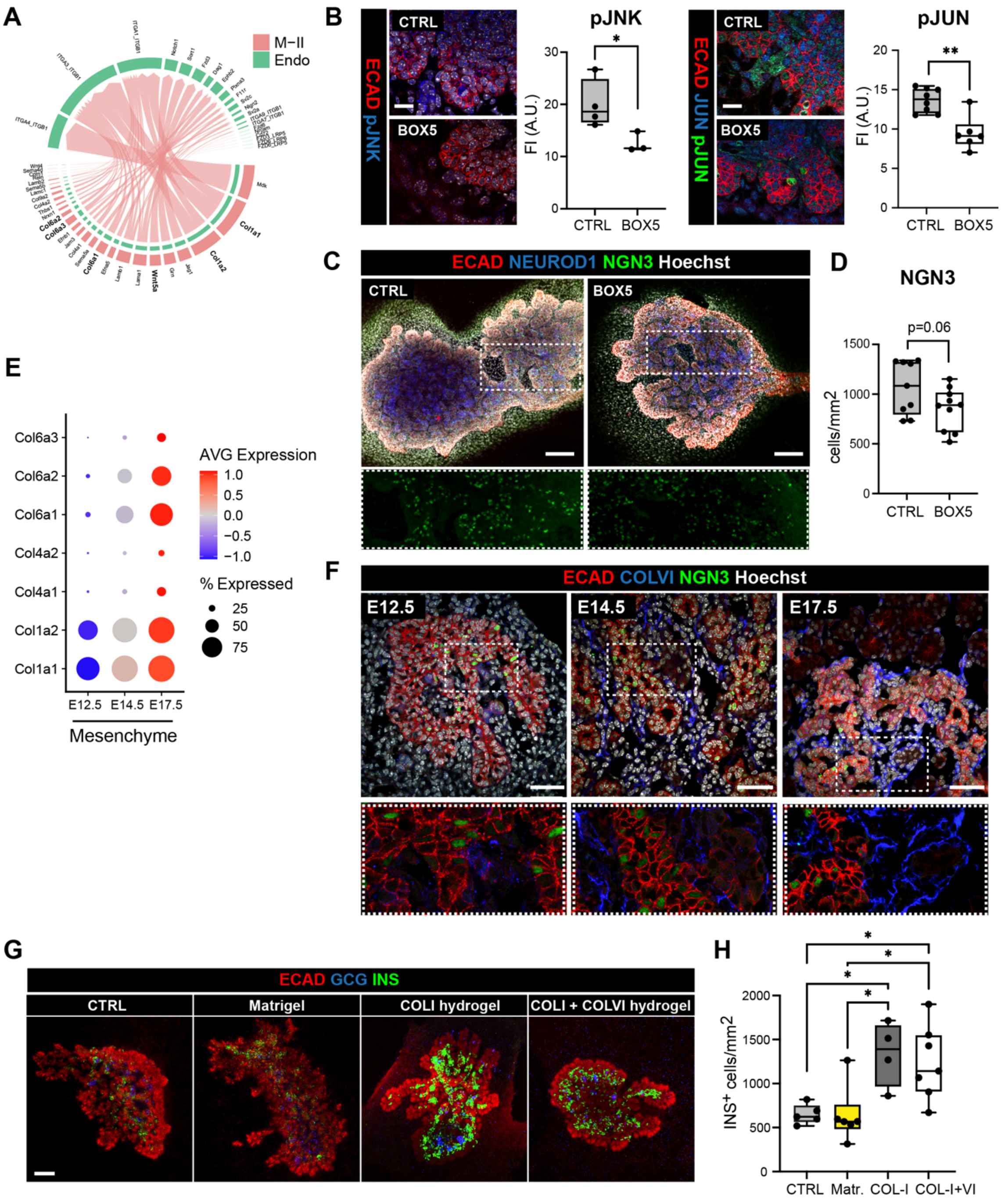

Fig. S8. Characterization of M-II signaling niche. (**A**) Chord diagram showing putative cell-cell communication between pancreatic M-II and endocrine cell types based on E12.5 Smart-seq2 data. Endocrine cells are the 'receiver' cell type, expressing receptors and M-II are the 'sender' cells, expressing ligands. In bold, candidate molecules that were validated. (**B**) Representative confocal images of mouse pancreatic explants treated for 2 days with BOX5 or left untreated as controls (CTRL) and immunostained with indicated antibodies. Scale bars, 25µm. Fluorescence intensity (FI) measurements of pJNK and pJUN, displayed as arbitrary units (A.U.). *p < 0.05; **p < 0.01. Two-tailed paired t-tests.

(**C**) Whole-mount IF analysis for Neurogenin (NGN3), NEUROD1 and E-cadherin (ECAD) on mouse pancreatic explants treated for 2 days with BOX5 or left untreated as controls (CTRL). Bottom panels show higher magnifications of the boxed region as NGN3 single channel (green). Hoechst was used as nuclear counterstain. Scale bars, 150μm. (**D**) Quantification of $NGN3^+$ cells in pancreatic explants. Cell counts were normalized to the average $ECAD^+$ epithelium area (mm^2). n= 9-10 explants per condition. Two-tailed paired t-tests. (**E**) Dot plot showing the expression level of selected collagen genes in pancreatic mesenchymal cells per embryonic stage. Colour bar indicates the mean of expression level. (**F**) Representative confocal images of E12.5, E14.5 and E17.5 pancreatic cryosections immunostained for the indicated markers. Bottom panels show higher magnifications of the boxed region without Hoechst. An increase in collagen deposition was observed in the developing pancreas between E12.5 and E17.5, with Collagen VI (COLVI) gradually accumulating around and inside the endocrine clusters ($NGN3^+$). Scale bars, 50 μm. (**G**) Representative whole-mount IF images of mouse pancreatic explants embedded in Matrigel, Collagen (COL) I, COLI and COLVI mix, or left unembedded (CTRL), stained for ECAD, GCG and INS. Murine pancreatic explants showed differences in their overall morphology and size according to the hydrogel composition. Explants embedded in Matrigel underwent extensive branching, whilst the ones in Collagen hydrogel showed a rounder morphology. Scale bar, 150 μm. (**H**) Quantification of INS-positive cells in embedded and unembedded pancreatic explants. Cell counts were normalized to the average $ECAD^+$ epithelium area (mm^2). n=5-6 explants per condition. *$p < 0.05$; One-way Anova test.

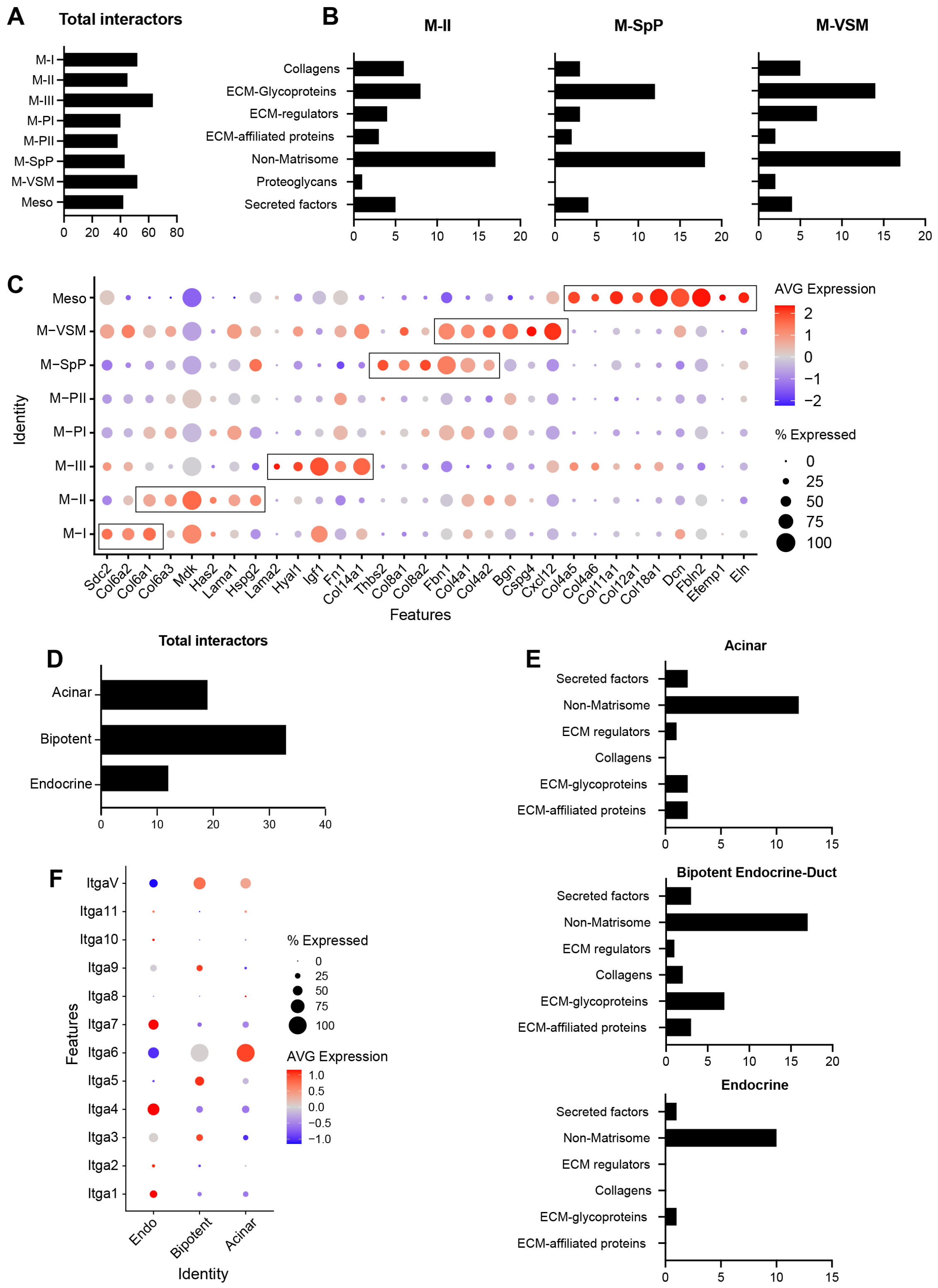

Fig. S9. Characterization of the pancreatic matrisome. (**A**) MatriCom *(41)* was used to mine the Smart-seq2 dataset and infer ECM–ECM and cell–ECM communication systems. Bar graph showing the number of total genes implicated in matrisome-matrisome and matrisome-non-matrisome communication types in each mesenchymal cell population. The full list of interactions can be accessed in table S7. (**B**) Bar graphs

showing the number of genes per matrisome category implicated in ECM communication in M-II, M-SpP and M-VSM populations. (**C**) Dot plot showing the expression level of a curated list of ECM-linked genes in mesenchymal clusters. Distinct ECM genes typify individual clusters: collagens (*Col8a1*, *Col8a2* in M-SpP, *Col6a1, Col6a2, Col6a3* in M-II, *Col18a1* in the Mesothelium), proteoglycans (*Bgn* in M-VSM, *Hspg2* in M-SpP and M-II or *Sdc2* in M-I), glycoproteins (*Fn1* in M-III) and secreted factors with an ECM modulating activity (*Igf1* in M-III or *Cxcl12* in M-VSM). Even though some of the *Col6* transcripts are detected in other mesenchymal sub-populations (*e.g.*, *Col6a1* in M-I), the MII sub-type expressed all primary Collagen VI transcripts (*Col6a1, Col6a2,* and *Col6a3*), which are responsible for producing the α1(VI), α2(VI), and α3(VI) chains, respectively. Specifically, the α3(VI) chain is necessary for successful assembly and secretion of the common form of Collagen VI *(68),* while α1(VI) and α2(VI) alone cannot form viable Collagen VI heterotrimers. Colour bar indicates the scaled mean of expression level. (**D**) Bar graph showing the number of genes implicated in matrisome-matrisome and matrisome-non-matrisome communication types in each epithelial cell population. Epithelial cells express fewer ECM-related genes compared to mesenchymal clusters. Within the epithelial compartment, bipotent endocrine-ductal cells exhibited the highest expression of ECM-related genes, whereas endocrine cells showed the lowest. (**E**) Bar graphs showing the number of genes per matrisome category implicated in ECM interactions in epithelial cell types. (**F**) Dot plot showing the expression level of Integrin alpha subunits able to dimerize with Integrin beta1 in each epithelial population. Colour bar indicates the scaled mean of expression level.

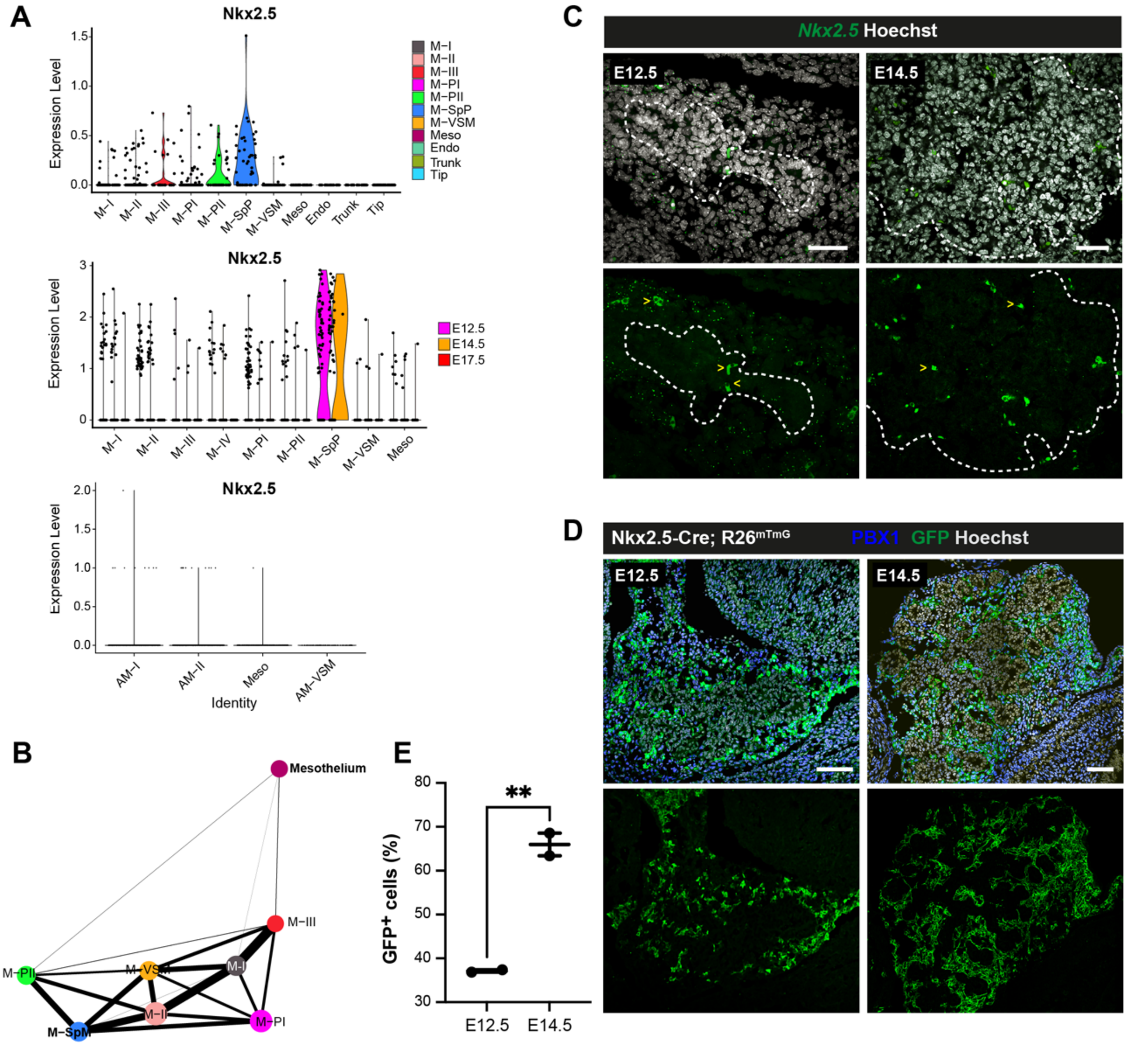

Fig. S10. Characterization of Nkx2.5 in mesenchymal cells. (**A**) Top panel, violin plot showing the expression of *Nkx2.5* in scRNAseq profiles from the Smart-seq2 dataset in mesenchymal and epithelial cell populations. Middle panel, violin plot showing the expression of *Nkx2.5* in scRNAseq profiles from the integrated dataset grouped by cluster and split by embryonic stage. Smart-seq2 dataset profiles were excluded from this analysis. Bottom panel, violin plot showing the expression of *Nkx2.5* in scRNAseq profiles from the integrated adult dataset grouped by cluster. *Nkx2.5* transcripts were not detected in adult pancreatic fibroblasts. (**B**) PAGA was applied to the E12.5 Smart-seq2 dataset to reconstruct the embryonic mesenchymal lineage. PAGA graph shows the connections between mesenchymal clusters and predicts the Mesothelium and M-SpP as opposite roots of the lineage. (**C**) Representative RNAscope images of E12.5 and E14.5 pancreatic tissue hybridized for *Nkx2.5*. Hoechst was used as nuclear counterstain. White dotted lines demarcate the pancreatic epithelium. Arrowheads indicate background staining in blood cells. Scale bar, 100µm. (**D**) Representative confocal microscopy images

of E12.5 and E14.5 pancreatic cryosections from Nkx2.5-Cre; $R26^{mTmG}$ embryos immunostained for indicated markers. Hoechst was used as nuclear counterstain. Scale bar, 150µm. (**E**) Graph showing the % of GFP-labelled cells in the pancreatic mesenchyme ($PBX1^{+}$) of Nkx2.5-Cre;$R26^{mTmG}$ embryos. The number of GFP^{+} cells increased during embryonic development despite the decrease in *Nkx2.5* levels of expression (fig. S10, A and B).

List of Supplementary Tables

File name: Table S1

Description: Differentially expressed genes of each cluster from the Smart-seq2 data set. Genes were calculated by comparing each cluster vs. all other clusters with Seurat's FindAllMarkers() function.

File name: Table S2

Description: Differentially expressed genes of each cluster from the integrated embryonic data set. Genes were calculated by comparing each cluster vs. all other clusters with Seurat's FindAllMarkers() function.

File name: Table S3

Description: Gene panels used for dRNA HybISS experiments. Each panel has its own tab.

File name: Table S4

Description: Tissue domain average cell type composition.

File name: Table S5

Description: Differentially expressed genes of each cluster from the adult mesenchyme data set. Genes were calculated by comparing each cluster vs. all other clusters with Seurat's FindAllMarkers() function.

File name: Table S6

Description: Differentially expressed genes of each cluster from the adult-embryonic integrated mesenchyme data set. Genes were calculated by comparing each cluster vs. all other clusters with Seurat's FindAllMarkers() function.

File name: Table S7

Description: Matrisome interactions between each cluster from the Smart-seq2 data set.

File name: Table S8

Description: List of primary and secondary antibodies, RNA probes, cytokines, media, and other reagents.

File name: Table S9

Description: Protocol, media and cytokines used for Beta Cell iPSC differentiation.
